# Network pharmacology and molecular dynamics simulation reveal antineoplastic potential of Antarctic sponge-derived suberitenones

**DOI:** 10.3389/fchem.2025.1545834

**Published:** 2025-05-27

**Authors:** Prasenjit Bhowmik, Rahul Mallick, Asim K. Duttaroy

**Affiliations:** ^1^ Department of Chemistry-BMC, Biochemistry, Disciplinary Domain of Science and Technology, Uppsala University, Uppsala, Sweden; ^2^ A.I. Virtanen Institute for Molecular Sciences, Faculty of Health Sciences, University of Eastern Finland, Kuopio, Finland; ^3^ Department of Nutrition, Institute of Medical Sciences, Faculty of Medicine, University of Oslo, Oslo, Norway

**Keywords:** network pharmacology, molecular docking, molecular dynamics simulation, suberitenone, CASP3, MAPK3, EGFR

## Abstract

**Introduction:**

More than a thousand new marine natural products have been isolated each year over the past ten years, and compared to synthetic compounds, the success ratio of approved marine drugs to the total number of reported potential marine natural products is extremely high. In a recent in vitro cytotoxicity test, 11 suberitenones–a class of oxidized sesterterpenes–were identified and shown to have low levels of cytotoxicity. This study focuses on the investigation of the anti-neoplastic ability of of these suberitenones through different in silico analysis.

**Methods:**

The study uses a variety of computational techniques, including quantitative structure–activity relationship (QSAR), ADMET, prediction of activity spectra for substances (PASS) prediction, network pharmacology, molecular docking, and molecular dynamics simulation.

**Results and discussion:**

The molecular docking showed that Suberitenone I, Secosuberitenone A, and Suberitenone J exhibited higher binding affinity of - 8.9, -9.4, and -8.8 kcal/mole against CASP3, MAPK3, and EGFR respectively which is further supported by molecular dynamics simulation analysis and can be considered for in vitro and in vivo investigation as potential antineoplastic agents.

## Graphical Abstract

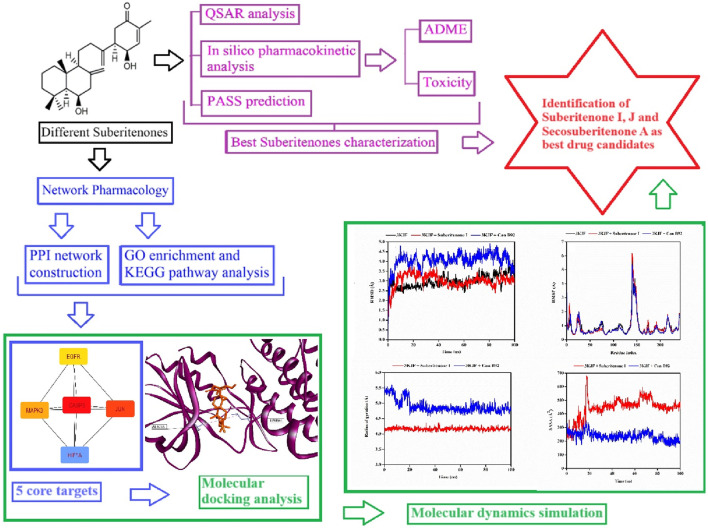

## Highlights

• Terpenoids were found to be effective against rapid tumor growth.

• Suberitenones, a class of oxidized sesterterpenes, demonstrated strong binding affinity for EGFR, MAPK3, and CASP3.

• Compared to the FDA-approved medication Osimertinib, two of the experimental Suberitenones demonstrated a greater binding affinity against EGFR.

• Molecular dynamics simulation suggests that Suberitenone I, Secosuberitenone A, and Suberitenone J may be used as antitumor agents against various targets.

## 1 Introduction

**FIGURE 1:**
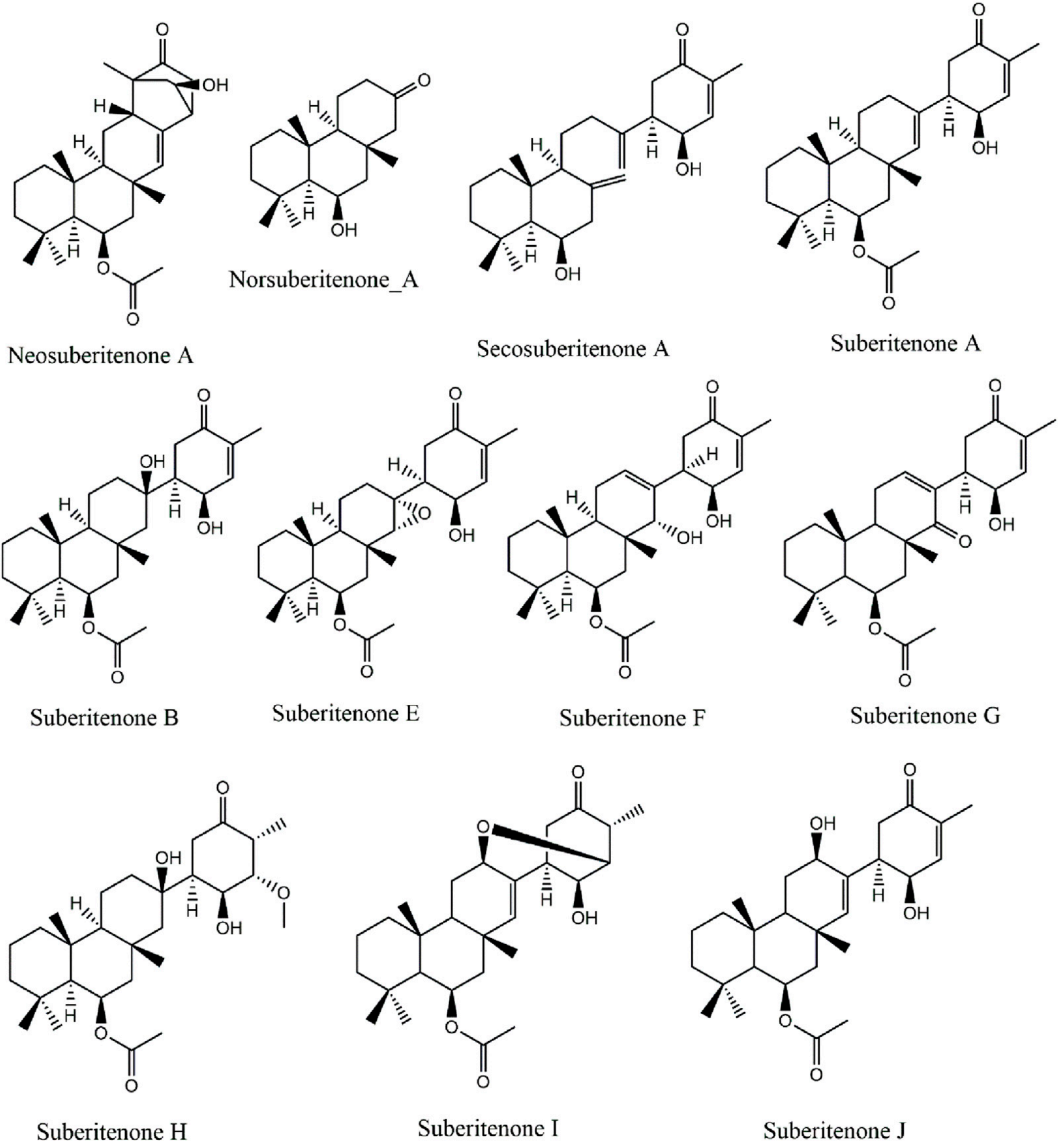
The structures of potential suberitenones.

Marine sponges found are sessile invertebrates known to be significant contributors of novel bioactive compounds. The natural products extracted from marine sponges are good antimicrobial, antitumor, and cytotoxic agents (Varijakzhan et al., 2021). Over the last decade, the number of new marine natural products isolated yearly is more than one thousand. The success ratio between the approved marine drugs and the total number of potential marine natural products reported is very high compared to synthetic compounds (Jiménez, 2018). The first marine isoprenes that Bergmann discovered during the 1930s–1940s from various microorganisms were steroidal terpenoids (Ebada and Proksch, 2012). Terpenoids and their many derivatives obtained from marine resources dominate the literature. Terpenoids are generally categorized according to the number of isoprene units building their parent terpene scaffold, such as hemiterpenoids (C_5_), monoterpenoids (C_10_), sesquiterpenoids (C_15_), diterpenoids (C_20_), sesterterpenoids (C_25_), triterpenoids (C_30_), tetraterpenoids (C_40_), and polyterpenoids (more than C_40_) (Liu et al., 2007; Wang et al., 2013). Sesterterpenoids, with a 25-carbon chain backbone, are one of the derivatives of marine terpenoids first reported in 1980 with antibiotic activity against *Streptomyces pyogenes* and *Staphylococcus aureus* (Dillp de Sllval and Scheuer, 1980; Ebada et al., 2010). All the reported subgroups, which are linear, monocarbocyclic, bicarbocyclic, tricarbocyclic, tetracarbocyclic, and miscellaneous sesterterpenoids, were found to exhibit significant cytotoxicity against tumor cells (Liu et al., 2007; Hog et al., 2012; Wang et al., 2013; Zhang and Liu, 2015). The suberitenones are a class of oxidized sesterterpenes from the genus Suberites of Antarctic sponges (Díaz-Marrero et al., 2004). Recently, Bracegirdle and researchers have characterized 11 suberitenones, of which only suberitenone A and suberitenone B were reported previously (Figure 1) (Bracegirdle et al., 2023). All the suberitenones were isolated from an Antarctic marine organism, and all showed a low cytotoxicity level against A549 cells (Bracegirdle et al., 2023).

**FIGURE 2:**
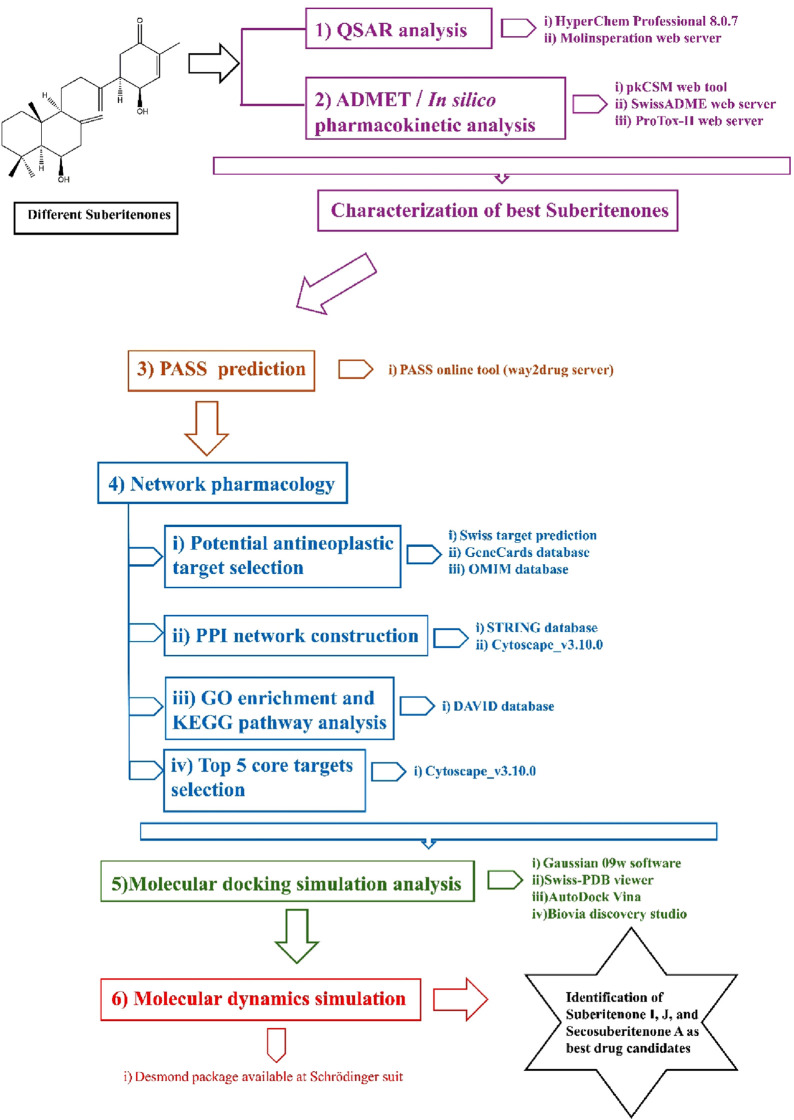
Graphical abstract of methods.

Cancer is a leading cause of death worldwide due to significant therapeutic obstacles, such as chemoresistance, toxicity, relapse, and metastasis. According to GlOBOCAN 2020, the number of recent cancer cases is 19.3 million, and nearly 10 million people died of cancer in 2020 (Ferlay et al., 2021). Cancer is a life-threatening disease that results from genetic mutations followed by the promotion of uncontrollable division of cells. The primary response of cells to exogenous or endogenous DNA damage is stimulating a repair system such as tumor suppressor gene P53 that can induce apoptosis according to the necessity and control cell cycle arrest to suppress subsequent damage (Kamran et al., 2022). However, when this response is disrupted, the process can lead to rapid tumor development. Some terpenoids, such as Abisilin^®^, were found to inhibit tumor growth *in vivo* and effectively stimulate apoptosis against different cancer cells (Li et al., 2015; Torgovnick and Schumacher, 2015; Nevzorova et al., 2017; Kuete et al., 2019). In this study, all suberitenones that have been isolated and characterized by Bracegirdle and researchers are investigated for their potential as antineoplastic agents through different *in silico* approaches such as network pharmacology, QSAR, ADMET analysis, molecular docking, and molecular dynamics (MD) simulation (Figure 2).

## 2 Materials and methods

### 2.1 Prediction of QSAR properties

Quantitative structure–activity relationship (QSAR) is a quantum chemical method that is used to find the connection between the molecular structure of a compound and its biological action. QSAR is frequently used in scientific drug development. Quantum structure–activity interactions are one of the most critical areas of chemometrics, which is used to link a specific biological or chemical activity to molecular characteristics derived from a molecular structure by establishing a mathematical relationship between molecular structure and properties using a mathematical statistical algorithm (Wang et al., 2021). The HyperChem Professional 8.0.7 program and a free cheminformatics software web tool, Molinspiration (Molinspiration Cheminformatics Free Web Services, https://Www.Molinspiration.Com, Slovensky Grob, Slovakia), were used to perform all the calculations (Molinspiration Cheminformatics, 2002). The partition coefficient (logP) and topological polar surface area (TPSA) values play a key role individually in measuring the cell permeability of the investigational compounds. Physical parameters such as mass, hydration energy, and polarizability are also helpful in measuring pharmacological properties (Matta, 2014; Isyaku et al., 2020; Mohapatra et al., 2021; Gholivand et al., 2022).

### 2.2 *In silico* pharmacokinetic analysis

Computer-aided *in silico* methods in pharmacokinetic studies are useful in filtering many drug candidates to a few with the best properties. Effective computer systems and theoretical chemistry approaches are utilized to calculate the physicochemical characteristics of candidate drugs. By combining pharmacokinetic processes in one model, the *in silico* pharmacokinetic studies help predict the possible behavior of candidate drugs *in vivo* (Hamidović et al., 2021).

#### 2.2.1 ADME and drug-like parameter prediction

Absorption, distribution, metabolism, and excretion (ADME) analysis studies the pharmacokinetic properties and features of drug-like compounds based on their molecular structures. Two online tools, pkCSM (PkCSM ADMET descriptors algorithm protocol, a freely accessible web server (http://Structure.Bioc.Cam.Ac.Uk/Pkcsm)) and SwissADME (http://www.swissadme.ch/) (Daina et al., 2017), were used to predict the physicochemical qualities, absorption, distribution, metabolism, elimination, and other pharmacokinetic properties of the investigational compounds, which is vital information in planning the procedure of clinical trials. After intestinal absorption, the distribution of drugs depends on different factors, including the blood–brain barrier (BBB) permeability (logBB), central nervous system (CNS) permeability, and the volume of distribution (VDss) (Han et al., 2019). Different cytochrome P (CYP) models, such as CYP2D6, CYP3A4, CYP1A2, CYP2C19, and CYP2C9, are used for substrate or inhibitor analysis to predict the metabolism and excretion of the substance based on the total clearance model (Han et al., 2019).

#### 2.2.2 Assessments of toxicity

One of the necessary steps in pharmacokinetic analysis is the prediction of toxicity of investigational compounds to identify their harmful effects on animals, humans, plants, or the environment. Although different animal models are used to determine the toxicity of drug candidates, *in vivo* animal tests are also restricted by poor prediction of drug safety in humans, time, cost, and ethical considerations (Raies and Bajic, 2016; Van Norman, 2019). Therefore, *in silico* toxicology analysis as an emerging field is considered valid when aiming to decrease the use of animal experiments (Hemmerich and Ecker, 2020). In this study, the quantitative and qualitative measurements of different classes of toxicity such as mutagenicity, carcinogenicity, acute toxicity, hepatotoxicity, and other features like lethal dose 50 (LD50), cardiac failure, heart block, and human ether-à-go-go-related gene (hERG) toxicity were accessed through the ProTox-II, and CardioToxCSM web servers (https://tox-new.charite.de/protox_II/) (Banerjee et al., 2018; Iftkhar et al., 2022).

### 2.3 PASS prediction

The two-dimensional (2D) structures of all suberitenones were drawn using ChemDraw Professional 16.0, and the 3D structures for energy optimization were prepared and converted to their SMILES file format using Chem3D 16.0 followed by the utilization of SMILES file to predict the biological activities using the prediction of activity spectra for substances (PASS) online tool (https://www.way2drug.com/PassOnline/index.php) (Lagunin et al., 2000). The PASS online tool was designed to provide 95% accurate predictions of a wide variety of biological activities (Parasuraman, 2011). The result is presented as Pa (probability for active compound) and interpreted at prediction threshold of Pa > 0.3, Pa > 0.5, and Pa > 0.7. When Pa > 0.7, the chance of determining the activity experimentally is high (Parasuraman, 2011).

### 2.4 Prediction of antineoplastic-related substances in suberitenones based on network pharmacology

#### 2.4.1 Screening of targets of suberitenones

To predict the information of suitable targets of all suberitenones, the Swiss Target Prediction database (http://www.swisstargetprediction.ch/, accessed on 16 July 2023) was used by importing SMILES format file of all suberitenones. The UniProt database (https://www.uniprot.org/, accessed on 16 July 2023) was used to search and validate the gene names by importing the target information. The screening condition was set by selecting “*Homo sapiens*” as the species, with probability > 0.

#### 2.4.2 Screening of potential targets for antineoplastic activity

The genes related to antineoplastic activity were searched in the GeneCards database (https://www.genecards.org/, accessed on 16 July 2023) for concise genomic-related information and the OMIM database (https://omim.org/, accessed on 16 July 2023) that is used for getting information related to human genes and genetic phenotypes. The targets from the GeneCards database were screened using the median screening method, and the antineoplastic targets from the OMIM database were selected for the removal of duplicates and to obtain the final list of targets related to antineoplastic effects.

#### 2.4.3 Protein interaction network construction (PPI)

A Venn diagram was drawn by importing the file, including the analysis of the intersection of the targets of all suberitenones and potential antineoplastic targets using the online website Venny 2.1.0 (https://bioinfogp.cnb.csic.es/tools/venny/index.html, accessed on 17 July 2023). STRING, a functional protein association networks database (https://cn.string-db.org/, accessed on 17 July 2023), was used to preliminarily obtain and export the protein interaction networking file of the antineoplastic effect in TSV file format. A protein interaction network was constructed by importing the TSV file format into Cytoscape_v3.10.0 for the antineoplastic targets (Shannon et al., 2003).

#### 2.4.4 GO enrichment and KEGG pathway analysis

The Gene Ontology (GO) enrichment analysis and the Kyoto Encyclopedia of Genes and Genomes (KEGG) enrichment analysis were performed by importing the obtained antineoplastic target proteins of all suberitenones into the Database for Annotation, Visualization, and Integrated Discovery(DAVID) database (https://david.ncifcrf.gov/, accessed on 17 July 2023). Entries with p < 0.05 were selected and sorted as significantly enriched GO entries or KEGG pathways.

### 2.5 Preparation of proteins and energy optimization of all suberitenones for molecular docking analysis

The complete sequence of CASP3, MAPK3, and EGFR was searched in the NCBI database, and three-dimensional (3D) structures were obtained from the PDB database (PDB codes: 3KJF, 6GES, and 6JXT, respectively) (Berman et al., 2000; Wang et al., 2010; Rao et al., 2019; Yan et al., 2020). Biovia Discovery Studio 2020 was utilized to remove unnecessary hetero atoms, and Swiss-PDB viewer (version 4.1.0), using the steepest descent algorithm, and the GROMOS96 43B1 force field were used to perform the energy minimization of the proteins and remove bad contacts in the protein structure (Ciucx and Peitsrh Urctrophuresis, 1997; Biovia, 2020). All three ligands were removed individually using Biovia Discovery Studio 2020 for redocking purposes. Gaussian 09w software was used to perform the energy optimization process, followed by the docking of all suberitenones by AutoDockVina in PyRx software (version 0.8) against all proteins individually (Dallakyan and Olson, 2015; Khaldan et al., 2024). The open-source software AutoDockVina and AutoDock Tools (ADT) of the MGL software package were utilized to convert all pdb files into the pdbqt format. In Vina Wizard (Version v1.2.3), the grid box was preserved at (i) X:39.9451, Y:12.4630, and Z:72.8688; (ii) X:22.9456, Y:−3.6424, and Z:11.4726; and (iii) X:−16.3197, Y:54.5071, and Z:9.1460 for (i) 3KJF, (ii) 6GES, and (iii) 6JXT, respectively. The structures with the highest binding scores were saved in pdb format using UCSF Chimera (candidate version 1.13) (Pettersen et al., 2004). Later, the non-bonding interactions between amino acids of receptor proteins and ligands were searched to determine the best binding pose using Biovia Discovery Studio 2020.

### 2.6 Protein–ligand stability analysis by molecular dynamics simulation

Molecular dynamics (MD) simulation analysis is used to check the structural stability of protein–ligand complexes in drug discovery. A 100-ns MD simulation was carried out to observe the consistency in the binding of 3KJF-Control B92, 3KJF-suberitenone I, 6GES-Control 6H3, 6GES-secosuberitenone A, 6JXT-suberitenone E, 6JXT-suberitenone J, and 6JXT-Control YY3 complexes using a Linux (Ubuntu-20.04.1 LTS) environment with an Intel Core i7-10700K processor CPU, 3200 MHzDDR4 RAM, and RTX 3080 DDR6 8704 CUDA core GPU following a previously reported protocol (Bhowmik et al., 2023). In the Desmond package available at the Schrödinger suit, the protein preparation wizard was used to preprocess protein–ligand complex structures generated from molecular docking (Bowers et al., 2006; Goyal and Goyal, 2020). To solve the system for each complex and maintain a specific volume, a simple point-charge (SPC) water model was used, followed by assigning an orthorhombic periodic boundary box shape with a distance (10 × 10 × 10 Å^3^). The salt concentration of the solvated system was maintained at 0.15 M by placing Na^+^ and Cl^−^ ions randomly, and the minimization and relaxation of the system were maintained using the OPLS3e force field (Roos et al., 2019). The constant pressure–constant temperature (NPT) ensemble was performed at 300.0 K temperature and 101,325 × 10^−5^ bar pressure, followed by the performance of the final production run with an energy of 1.2 eV after the relaxation of the system using 100 picoseconds recording interval for each complex (Ahammad et al., 2021; Bouback et al., 2021). Finally, the calculated root mean square deviation (RMSD), root mean square fluctuation (RMSF), R_g_, solvent accessible surface area (SASA), and protein–ligand contact analysis data were analyzed to get a notion of possible changes in vibrant binding behavior of the aforementioned protein–ligand complexes in various poses under specific physiological environments compared to the protein in the apo state.

## 3 Results and discussion

### 3.1 Prediction of QSAR properties

Various properties, including mass, HE, MR, LogP value, TPSA, and numbers of H bond donors and acceptors, are investigated to understand the possible transportation efficiency and structural flexibility of the drug candidates. Drugs with a molecular weight below 450 atomic mass units (amu) can have good blood–brain barrier (BBB) penetration (van de Waterbeemd et al., 1998). Based on the statistical distribution, HE values less than −5 kcal/mol and MR scores between 40 Å^3^ and 130 Å^3^ are preferable for developing successful drug candidates (Zafar and Reynisson, 2016; Akash et al., 2023). The HE values given in Table 1 are higher than −5 kcal/mol; however, one negative factor alone does not determine the final potential of these candidates. The polar surface area indicates the oral absorption, oral bioavailability, intestinal permeability, and central nervous system (CNS) penetration capability of small molecule drugs (Clark, 2011). A positive LogP value represents hydrophobicity, while hydrophilicity is indicated by a negative LogP value (Islam et al., 2019). As drugs with high hydrophobicity or high hydrophilicity are not good in transportation through the bloodstream or efficient in binding to the target, respectively, a LogP value in the moderate range is advantageous in both cases. Drugs with good intestinal permeability and CNS penetration ability have a TPSA of less than 140 Å^2^ and 60 Å^2^, respectively (Pajouhesh and Lenz, 2005; Shityakov et al., 2013). According to the TPSA values given in Table 1, all investigational compounds are good for intestinal permeation, while only neosuberitenone A, norsuberitenone A, secosuberitenone A, and suberitenone A can be considered good for CNS penetration as well. The total number of oxygen and nitrogen atoms (nON), the total number of -OH and -NH groups (nOHNH), and the number of rotatable bonds (nrotb) less than 7, 3, and 8, respectively, are characteristics of drugs with more structural flexibility and good capability of CNS penetration (Pajouhesh and Lenz, 2005). Although the CNS permeability of all investigational compounds is indicated by other properties like nON, nOHNH, and nrotb, the TPSA values show that only four of the compounds can interpenetrate and diffuse through the CNS. Overall, all investigational compounds can be considered good drug candidates according to the QSAR studies. Of the experimental drug candidates, only neosuberitenone A, norsuberitenone A, secosuberitenone A, and suberitenone A should be regarded as ideal choices due to their capacity to penetrate and diffuse through the CNS as well as penetrate the intestine according to all parameters.

**TABLE 1:** QSAR studies of the suberitenones.

Properties	Neosuberitenone A	Norsuberitenone A	Secosuberitenone A
Mass (amu)	428.61	278.44	386.58
HE (Kcal/mol)	−3.13	0.037	−2.42
MR (A3)	107.07	77.82	89.20
Pol (A3)	47.70	32.20	45.11
LogP	5.27	3.68	5.02
TPSA (A2)	63.60	37.30	57.53
Volume	424.77	290.96	399.01
nHA	31	20	28
nON	4	2	3
nOHNH	1	1	2
Nviolations	1	0	1
Nrotb	2	0	4
Enzyme inhibitor	0.60	0.63	0.55
Nuclear receptor ligand	0.65	0.58	0.76
G protein-coupled receptor (GPCR) ligand	0.06	0.14	0.13
Ion channel modulator	0.03	0.18	0.3
Protease inhibitor	−0.02	−0.01	0.01
Kinase inhibitor	−0.52	−0.57	−0.61

Properties	Suberitenone A	Suberitenone B	Suberitenone E
Mass (amu)	428.61	446.63	444.61
HE (Kcal/mol)	−0.69	−2.75	−3.95
MR (A3)	101.72	110.47	105.99
Pol (A3)	48.22	49.11	48.56
LogP	5.49	4.73	4.93
TPSA (A2)	63.60	83.83	76.13
Volume	429.70	443.61	433.76
nHA	31	32	32
nON	4	5	5
nOHNH	1	2	1
Nviolations	1	0	0
Nrotb	3	3	3
Enzyme inhibitor	0.60	0.48	0.57
Nuclear receptor ligand	0.77	0.50	0.68
G protein-coupled receptor (GPCR) ligand	0.12	0.23	0.18
Ion channel modulator	0.24	0.16	0.26
Protease inhibitor	−0.00	0.05	0.20
Kinase inhibitor	−0.64	−0.65	−0.59

Properties	Suberitenone F	Suberitenone G	Suberitenone H
Mass (amu)	444.61	442.60	478.67
HE (Kcal/mol)	−4.34	−3.43	−3.13
MR (A3)	102.90	98.88	124.07
Pol (A3)	48.92	48.37	51.78
LogP	4.58	4.39	4.38
TPSA (A2)	83.83	80.67	93.07
Volume	437.74	431.88	475.39
nHA	32	32	34
nON	5	5	6
nOHNH	2	1	2
Nviolations	0	0	0
Nrotb	3	3	4
Enzyme inhibitor	0.50	0.55	0.53
Nuclear receptor ligand	0.55	0.60	0.51
G protein-coupled receptor (GPCR) ligand	0.14	0.02	0.28
Ion channel modulator	0.24	0.08	0.12
Protease inhibitor	0.01	−0.04	0.24
Kinase inhibitor	−0.61	−0.070	−0.42

Properties	Suberitenone I	Suberitenone J	
Mass (amu)	444.61	444.61	
HE (Kcal/mol)	−4.38	−6.00	
MR (A3)	108.39	103.05	
Pol (A3)	48.34	48.92	
LogP	4.72	4.58	
TPSA (A2)	72.84	83.83	
Volume	434.11	437.74	
nHA	32	32	
nON	5	5	
nOHNH	1	2	
Nviolations	0	0	
Nrotb	2	3	
Enzyme inhibitor	0.67	0.57	
Nuclear receptor ligand	0.73	0.72	
G protein-coupled receptor (GPCR) ligand	0.13	0.09	
Ion channel modulator	0.16	0.20	
Protease inhibitor	0.20	0.04	
Kinase inhibitor	−0.45	−0.61	

^a^ HE = hydration energy; LogP = octanol–water partition coefficient; MR = molecular refractivity; nHA = number of heavy atoms; nOHNH = total number of -OH and -NH groups; nON = total number of oxygen and nitrogen atoms; Nrtob = number of rotatable bonds; Pol = polarizability; TPSA = topological polar surface area.

### 3.2 Prediction of pharmacokinetic properties

A percentage of gastrointestinal absorption of small molecules below 30% is considered poorly absorbed (Kalantzi et al., 2006). All investigational compounds showed a high absorption percentage by the human intestine, which is given in Table 2. The threshold level for the steady-state volume of distribution (VDss), the BBB permeability (LogBB), and the CNS index (Log PS) are 0.45, 0.3, and −2, respectively (Pires et al., 2015; Han et al., 2019; Speciale et al., 2021). All compounds are suitable for BBB permeation; however, neosuberitenone A, secosuberitenone A, suberitenone I, and suberitenone J might be less effective in the case of CNS permeation. More than 90% of drugs that have passed the first phase of metabolism are bio-transformed by cytochrome P450 (CYP) (1A2, 2C9, 2C19, 2D6, and 3A4). The isoforms 3A4 and 2D6 alone account for the metabolism of more than 50% and about 25% of all drugs in the market, respectively (Wang et al., 2009; Zanger and Schwab, 2013; Teo et al., 2015; Rodrigues-Junior et al., 2020). All compounds, excluding norsuberitenone A, suberitenone B, and suberitenone F, can be metabolized by CYP3A4.

**TABLE 2:** ADME analysis of the suberitenones.

Model name	Neosuberitenone A	Norsuberitenone A	Secosuberitenone A
Absorption			
Intestinal absorption (human) (% absorbed)	99.37	95.95	95.42
Distribution			
VDss (human) (Log L/kg)	0.27	0.38	0.03
BBB permeability (Log BB)	−0.23	−0.01	−0.33
CNS permeability (Log PS)	−1.6	−2.35	−1.75
Metabolism			
CYP2D6 substrate	No	No	No
CYP3A4 substrate	Yes	No	Yes
CYP3A4 inhibitor	No	No	No
CYP1A2 inhibitor	No	No	No
CYP2C19 inhibitor	No	No	No
CYP2C9 inhibitor	No	No	No
Excretion			
Total clearance (logmL.min^−1^.kg^−1^)	0.12	0.62	0.71
Other properties			
Lipinski rule	Yes; 1 violation: MLOGP>4.15	Yes; 0 violation	Yes; 0 violation
PAINS (alert)	0	0	0
Brenk (alert)	1 alert: isolated_alkene	0	1 alert: isolated_alkene

Model name	Suberitenone A	Suberitenone B	Suberitenone E
Absorption			
Intestinal absorption (human) (% absorbed)	96.36	95.32	97.83
Distribution			
VDss (human) (Log L/kg)	0.15	−0.1	0.12
BBB permeability (Log BB)	−0.04	0.26	−0.16
CNS permeability (Log PS)	−0.27	−3.04	−2.87
Metabolism			
CYP2D6 substrate	No	No	No
CYP3A4 substrate	Yes	No	Yes
CYP3A4 inhibitor	No	No	Yes
CYP1A2 inhibitor	No	No	No
CYP2C19 inhibitor	No	No	No
CYP2C9 inhibitor	No	No	No
Excretion			
Total clearance (LogmL.min^−1^.kg^−1^)	0.35	0.33	0.26
Other Properties			
Lipinski rule	Yes; 1 violation: MLOGP>4.15	Yes; 0 violation	Yes; 0 violation
PAINS (alert)	0	0	0
Brenk (alert)	1 alert: isolated_alkene	0	1 alert: Three-membered_heterocycle

Model name	Suberitenone F	Suberitenone G	Suberitenone H
Absorption			
Intestinal absorption (human) (% absorbed)	99.13	99.96	81.82
Distribution			
VDss (human) (Log L/kg)	0.09	0.12	−0.06
BBB permeability (Log BB)	0.22	0.08	0.006
CNS permeability (Log PS)	−2.72	−2.69	−2.77
Metabolism			
CYP2D6 substrate	No	No	No
CYP3A4 substrate	No	Yes	Yes
CYP3A4 inhibitor	No	No	Yes
CYP1A2 inhibitor	No	No	No
CYP2C19 inhibitor	No	No	No
CYP2C9 inhibitor	No	No	No
Excretion			
Total clearance (LogmL.min^−1^.kg^−1^)	0.41	0.38	0.41
Other Properties			
Lipinski rule	Yes; 0 violation	Yes; 0 violation	Yes; 0 violation
PAINS (alert)	0	0	0
Brenk (alert)	1 alert: isolated_alkene	0	0

Model name	Suberitenone I	Suberitenone J
Absorption			
Intestinal absorption (human) (% absorbed)	97.21	94.66	
Distribution			
VDss (human) (Log L/kg)	0.02	−0.09	
BBB permeability (Log BB)	−0.19	−0.23	
CNS permeability (Log PS)	−1.68	−1.78	
Metabolism			
CYP2D6 substrate	No	No	
CYP3A4 substrate	Yes	Yes	
CYP3A4 inhibitor	No	No	
CYP1A2 inhibitor	No	No	
Metabolism			
CYP2C19 inhibitor	No	No	
CYP2C9 inhibitor	No	No	
Excretion			
Total clearance (LogmL.min^−1^.kg^−1^)	0.26	0.41	
Other properties			
Lipinski rule	Yes; 0 violation	Yes; 0 violation	
PAINS (alert)	0	0	
Brenk (alert)	1 alert: isolated_alkene	1 alert: isolated_alkene	

All investigational compounds except neosuberitenone A and suberitenone A tend to fulfill the Lipinski “Rule of Five” criteria. To identify problematic fragments within the structure of all investigational suberitenones, pan assay interference compounds (PAINS), a.k.a. frequent hitters or promiscuous compounds, and Brenk’s structural alert are analyzed. The substructures of any compounds that give false positive biological output in assays are identified by PAINS, and Brenk provides a list of 105 fragments responsible for poor pharmacokinetic properties (Brenk et al., 2008; Baell and Holloway, 2010). According to the Brenk alert, the presence of the isolated alkene in neosuberitenone A, secosuberitenone A, suberitenone A, suberitenone F, suberitenone I, and suberitenone J and the presence of the three-membered heterocycle in suberitenone E can be disadvantageous in the case of having good ADME properties.

### 3.3 Toxicity analysis

The late rise of severe and unfavorable side effects after using different small molecules against different targets is one of the most important factors for many research projects not reaching the final stage. In many cases, small molecule drugs can bind to a minimum of 6–11 off-targets on average with weak binding affinity, excluding their intended pharmacological target, leading to adverse side effects such as failure of major organs (Metz and Hajduk, 2010; Whitebread et al., 2016; Peón et al., 2017). The study of the possible toxicity of the investigational compounds can help identify the secondary pharmacology of those compounds (Whitebread et al., 2016). According to the toxicity analysis data given in Table 3, suberitenones other than suberitenone A, suberitenone E, suberitenone I, and suberitenone J showed good toxicity properties. Some of them may be carcinogenic or exhibit toxicity against the immune system. According to the lethal dose 50 (LD50) values, secosuberitenone A and suberitenone H will be the most tolerated inside the human body compared to other suberitenones. Only suberitenone E was found to be toxic in case of both cardiac failure and heart block. Suberitenone J has no possibility of causing cardiac failure, heart block, or hERG toxicity.

**TABLE 3:** Toxicity analysis of suberitenones.

Compound name	Toxicity class	LD50 (mg kg^−1^)	Cytotoxicity and mutagenicity
Neosuberitenone A	4	2,000	Inactive
Norsuberitenone A	5	5,100	Inactive
Secosuberitenone A	6	9,000	Inactive
Suberitenone A	3	200	Inactive
Suberitenone B	5	3,300	Inactive
Suberitenone E	3	79	Inactive
Suberitenone F	5	2,450	Inactive
Suberitenone G	5	3,300	Inactive
Suberitenone H	6	50,100	Inactive
Suberitenone I	2	34	Inactive
Suberitenone J	3	200	Inactive

Compound name	Immuno-toxicity	Carcinogenicity	Hepato-toxicity
Neosuberitenone A	Active	Inactive	Inactive
Norsuberitenone A	Inactive	Inactive	Inactive
Secosuberitenone A	Active	Active	Inactive
Suberitenone A	Active	Inactive	Inactive
Suberitenone B	Active	Inactive	Inactive
Suberitenone E	Active	Active	Inactive
Suberitenone F	Active	Active	Inactive
Suberitenone G	Active	Inactive	Inactive
Suberitenone H	Active	Inactive	Inactive
Suberitenone I	Active	Inactive	Inactive
Suberitenone J	Active	Active	Inactive

Compound name	Cardiac failure	Heart block	hERG toxicity
Neosuberitenone A	Toxic	Safe	Safe
Norsuberitenone A	Toxic	Safe	Safe
Secosuberitenone A	Safe	Safe	Toxic
Suberitenone A	Safe	Safe	Safe
Suberitenone B	Safe	Safe	Safe
Suberitenone E	Toxic	Toxic	Safe
Suberitenone F	Toxic	Safe	Safe
Suberitenone G	Toxic	Safe	Safe
Suberitenone H	Toxic	Safe	Safe
Suberitenone I	Toxic	Safe	Safe
Suberitenone J	Safe	Safe	Safe

### 3.4 Biological activities using PASS prediction

PASS software can predict the probability of different small or drug-like molecules belonging to a specific class of bioactive compounds based on the structure–activity relationship (Lagunin et al., 2000). The PASS Online tool can predict more than 3,678 pharmacological effects, modes of action, and other biological properties of compounds such as carcinogenicity, teratogenicity, etc (Marwaha et al., 2007; Matin et al., 2016). According to the PASS prediction given in Table 4 and considering P_a_ > 8, all investigational compounds, excluding suberitenone F, are good antineoplastic agents.

**TABLE 4:** Data of pass prediction.

Compound name	Antineoplastic properties (P_a_)	Other properties (P_a_)	
Neosuberitenone A	Antineoplastic 0.844	Apoptosis agonist 0.794	Ecdysone 20-monooxygenase inhibitor 0.774
Norsuberitenone A	Antineoplastic 0.846	Testosterone 17 beta dehydrogenase (NADP+) inhibitor 0.922	CYP2J substrate 0.920
Secosuberitenone A	Antineoplastic 0.809	Transcription factor NF kappa B stimulant 0.849	Antieczematic 0.846
Suberitenone A	Antineoplastic 0.819	Apoptosis agonist 0.816	CYP2J substrate 0.819
Suberitenone B	Antineoplastic 0.861	Ecdysone 20-monooxygenase inhibitor 0.782	Caspase 3 stimulant 0.763
Suberitenone E	Antineoplastic 0.873	Chemopreventive 0.740	Caspase 3 stimulant 0.744
Suberitenone F	Antineoplastic 0.749	Caspase 3 stimulant 0.850	CYP2J substrate 0.789
Suberitenone G	Antineoplastic 0.817	Caspase 3 stimulant 0.856	Apoptosis agonist 0.807
Suberitenone H	Antineoplastic 0.894	CYP2H substrate 0.781	Ecdysone 20-monooxygenase inhibitor 0.730
Suberitenone I	Antineoplastic 0.848	Hepatic disorder treatment 0.825	Glyceryl-ether monooxygenase inhibitor 0.736
Suberitenone J	Antineoplastic 0.807	Apoptosis agonist 0.837	Caspase 3 stimulant 0.824

### 3.5 Suberitenone targets and antineoplastic targets

**FIGURE 3:**
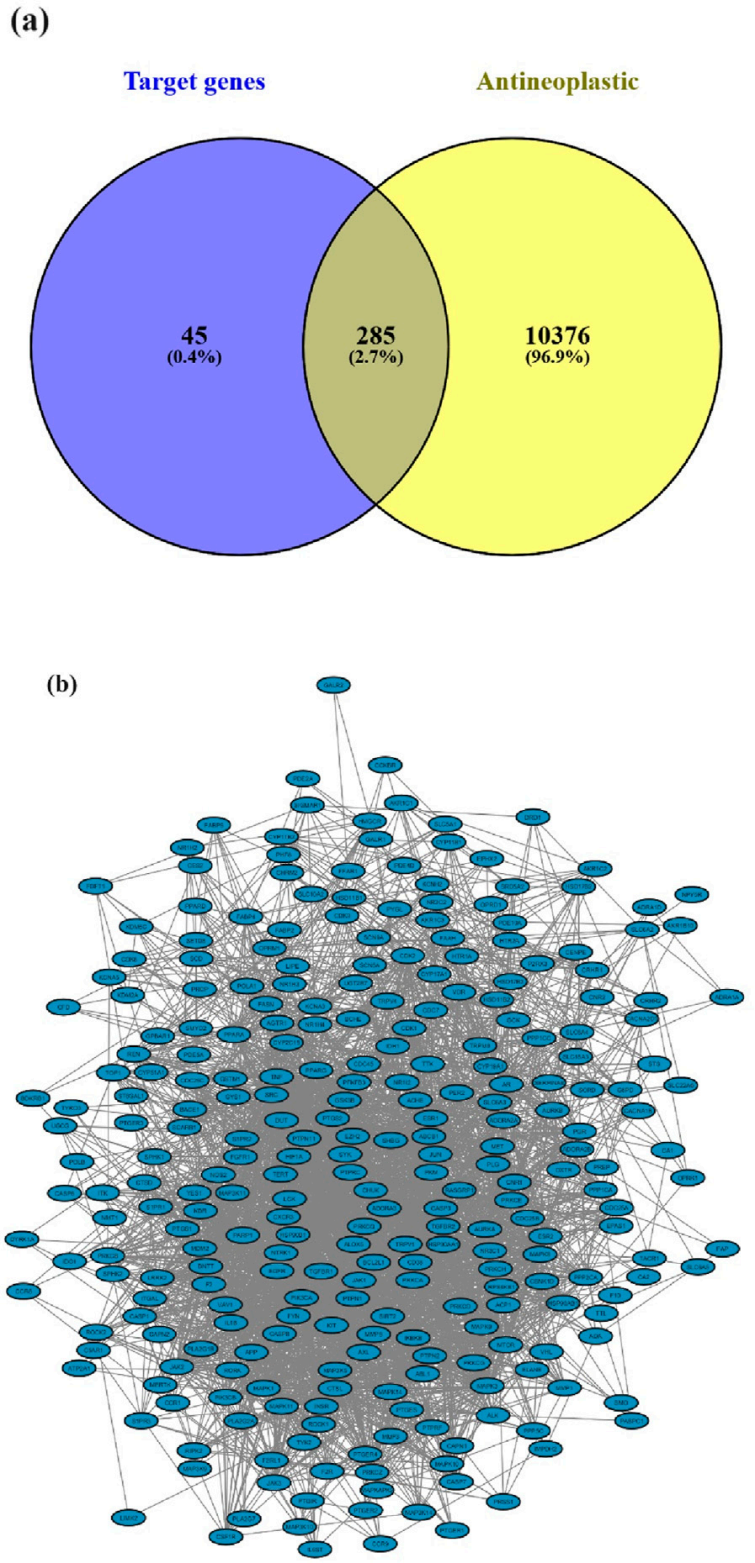
**(a)** Potential antineoplastic targets of suberitenones; **(b)** target protein PPI network for the antineoplastic activity of suberitenones.

All investigated suberitenones were searched, and the target gene names were based on the Swiss Target Prediction database platform. The predicted target gene names were confirmed and collected using the UniProt database. A total of 330 target genes of 11 candidates were retrieved after deleting the duplicates or invalid targets. The Genecards and OMIM databases were used to search and screen antineoplastic-related targets, and a total of 10,661 targets were obtained. The candidate compounds and disease targets were mapped (Figure 3a), resulting in 285 intersecting potential antineoplastic targets (Supplementary Table S1).

### 3.6 Protein–protein interaction (PPI) network construction for the potential antineoplastic targets of suberitenones

The potential antineoplastic targets were uploaded to the STRING database to construct a PPI network (Figure 3b). The number of nodes and edges in the PPI were 285 and 3,183, respectively, with two targets (TACR2 and ZAK) removed later for not being involved in the protein interactions.

### 3.7 Enrichment of antineoplastic Gene Ontology (GO) function and KEGG pathway analysis in all investigational suberitenones

**FIGURE 4:**
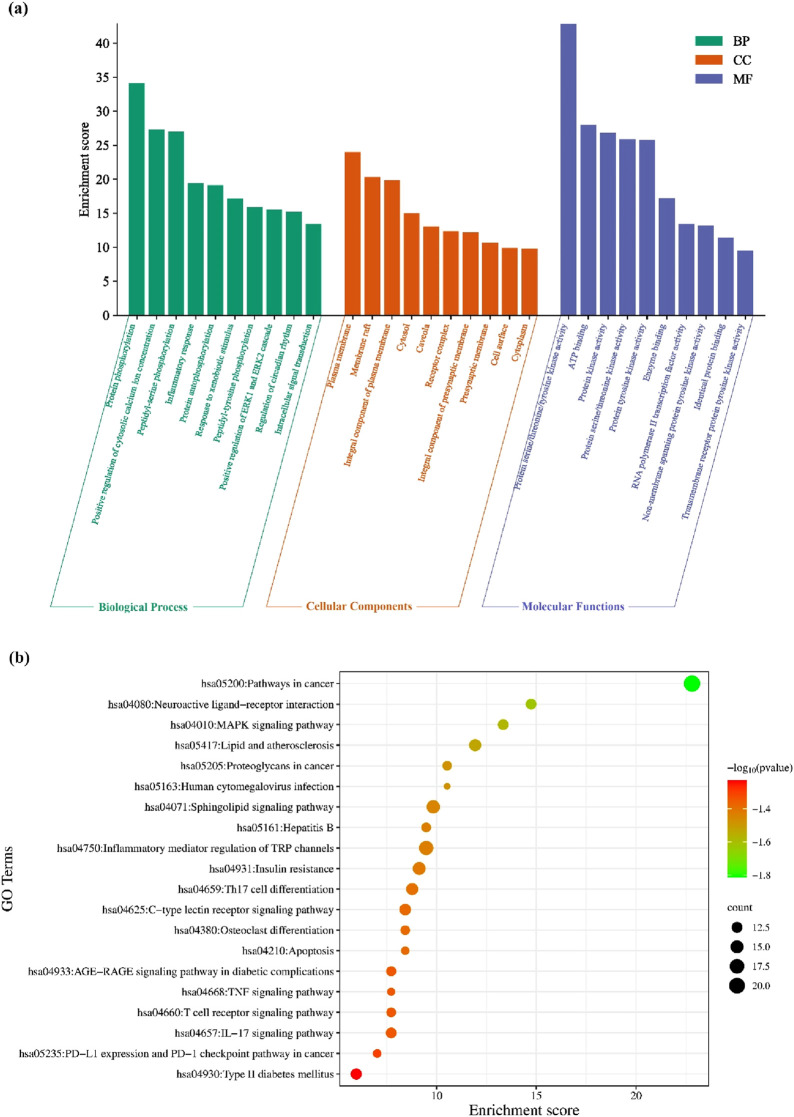
**(a)** Analysis of antineoplastic activity in investigational suberitenones by GO functional enrichment; **(b)** enrichment analysis of the antineoplastic KEGG pathway in investigational suberitenones.

The GO functional enrichment analysis of the potential antineoplastic targets was carried out using the DAVID database. A total of 1,083 pathways were obtained, including 765 biological processes (BP), 100 cellular components (CC), and 218 molecular functions (MF). With p < 0.005 as the screening condition, the top 20 counts from the result were used for the GO functional enrichment map (Figure 4a). Protein phosphorylation, positive regulation of cytosolic calcium ion concentration, inflammatory response, positive regulation of ERK1 and ERK2 cascade, regulation of circadian rhythm, etc., are the main biological processes that include the potential antineoplastic targets of the investigational compounds. The plasma membrane, membrane raft, cytosol, presynaptic membrane with its integral component, etc., are the cellular components. Protein serine/threonine/tyrosine kinase activity, ATP binding, RNA polymerase II transcription factor activity, non-membrane spanning protein tyrosine kinase activity, ligand-activated sequence-specific DNA binding, etc., are the functions in the molecular level that involve the targets.

The KEGG pathway enrichment analysis of 285 potential antineoplastic targets of the investigational compounds was analyzed. The result indicated the involvement of different pathways, such as pathways in cancer, inflammatory mediator regulation of transient receptor potential (TRP) channels, and sphingolipid signaling pathway. The KEGG pathway map was constructed using the top 20 counts with p < 0.005 as the screening condition (Figure 4b).

### 3.8 Molecular docking analysis

**FIGURE 5:**
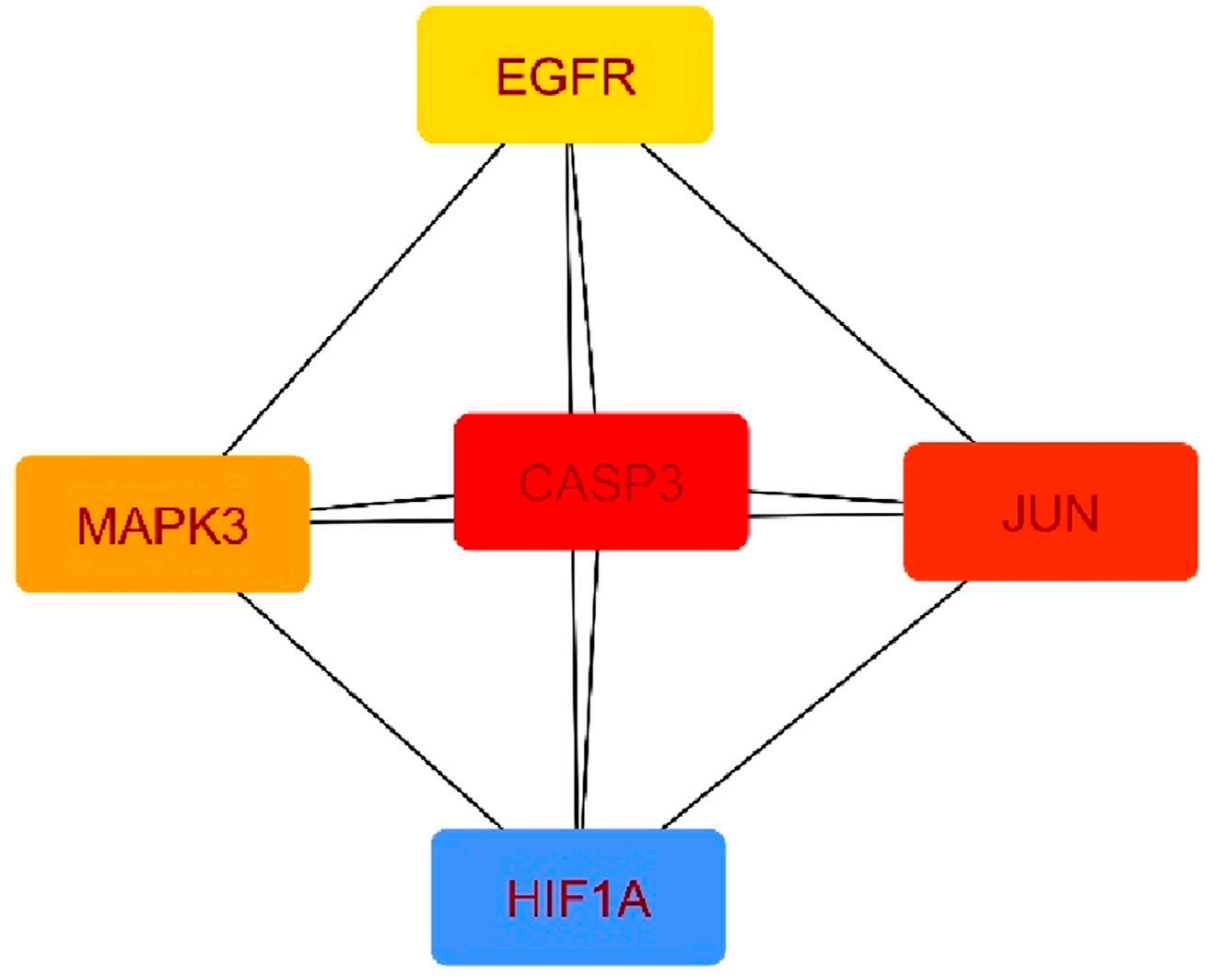
Top five core target maps.

**FIGURE 6:**
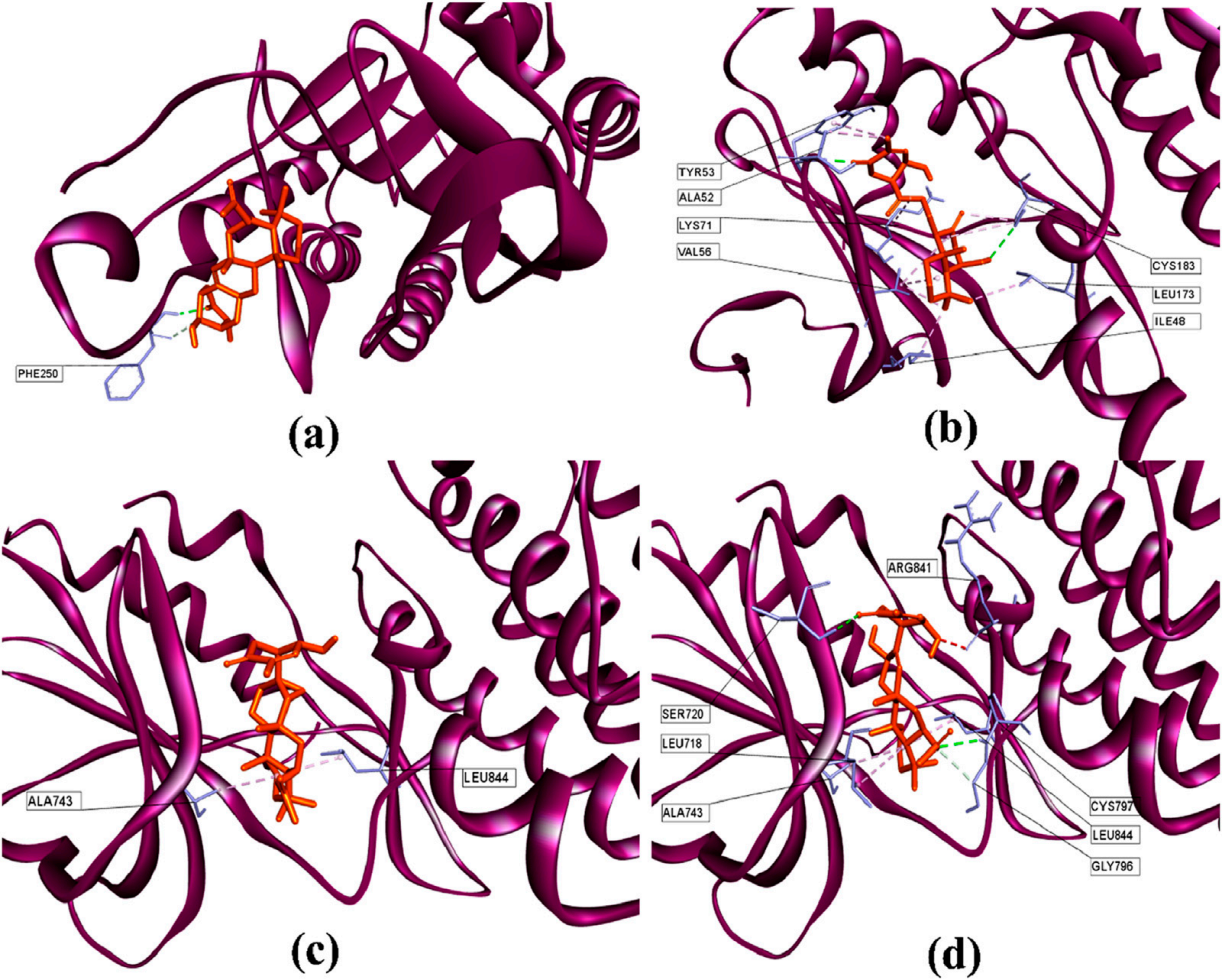
3D structures of **(a)** suberitenone I against the catalytic site of CASP3 (PDB: 3KJF); **(b)** secosuberitenone A against the catalytic site of MAPK3 (PDB: 6GES); **(c)** suberitenone E, and **(d)** suberitenone J bound to the catalytic site of EGFR (PDB: 6JXT).

Using the Cytohubba plugin and the maximal clique centrality (MCC) algorithm in Cytoscape_v3.10.0, the top five core targets were selected from the PPI network that might play an essential role in the antineoplastic ability of the investigational compounds (Figure 5 **)**. According to the molecular docking analysis (Figure 6) of the investigational suberitenones against the top five core targets, all suberitenones showed good combination ability only with CASP3 (PDB ID: 3KJF), MAPK3 (PDB ID: 6GES), and EGFR (PDB ID: 6JXT) based on the binding energy (Table 5).

**TABLE 5:** Binding energy of all suberitenones against different target proteins.

Targets with binding energy (kcal/mol)			
Compound	CASP3	JUN	HIF1A
Neosuberitenone A	−7.6	−6	−6.1
Norsuberitenone A	−6.8	−5	−5.1
Secosuberitenone A	−6.7	−5.9	−6.1
Suberitenone A	−7.5	−6	−6.3
Suberitenone B	−7.6	−6.1	−5.6
Suberitenone E	−8.1	−5.6	−5
Suberitenone F	−8.0	−5.5	−5.7
Suberitenone G	−8.0	−6	−5.5
Suberitenone H	−7.5	−5.3	−5.4
Suberitenone I	−8.9	−5.8	−5.7
Suberitenone J	−7.9	−6.3	−5.7

Compound	MAPK3	EGFR	
Neosuberitenone A	−7.7	−8.4	
Norsuberitenone A	−7.5	−7.6	
Secosuberitenone A	−9.4	−8.4	
Suberitenone A	−8.5	−7.9	
Suberitenone B	−8	−7.9	
Suberitenone E	−8.6	−8.8	
Suberitenone F	−8.4	−8	
Suberitenone G	−7.9	−7.7	
Suberitenone H	−7.3	−7.2	
Suberitenone I	−7.4	−8	
Suberitenone J	−7.6	−8.8	

Caspases are cysteine proteases essential in controlling cell death mediated by apoptosis, pyroptosis, necroptosis, and autophagy (Shalini et al., 2015). Among them, caspase 3, upon activation by initiator caspase 8 or caspase 9, leads to apoptosis of many critical proteins within the cell (Zhou et al., 2018). Caspase 3 provides a proangiogenic microenvironment for tumor growth and promotes tumor repopulation through the pancreatic signaling pathway after radiotherapy (Huang et al., 2011; Feng et al., 2017). Mitogen-activated protein kinase 3 (MAPK3) is a critical signaling molecule in the ERK/MAPK pathway. MAPK3 participates in cell proliferation and apoptosis through the phosphorylation of cytoplasmic proteins and activating several nuclear transcription factors such as c-Jun and c-fos (McGinnis et al., 2015; Taherkhani et al., 2023). Overexpression of MAPK3 has been associated with initiation, development, cancer cell migration, and drug resistance in different carcinogenic cells (Cao et al., 2019). EGFR is a crucial oncogene that can initiate the cascade of downstream signaling and is altered most frequently in carcinogenesis (Santarius et al., 2010; Yarden and Pines, 2012). Mutations in EGFR have been found often in non-small cell lung cancer (NSCLCs) and glioblastoma cells and have shown resistance to anti-EGFR therapies (Thomas and Weihua, 2019). The caspase 3 inhibitor B92, an MAPK3 substrate (PDB ligand code 6H3), and an FDA-approved third-generation epidermal growth factor receptor (EGFR) tyrosine kinase inhibitor (TKI) drug osimertinib were docked against 3KJF, 6GES, and 6JXT, respectively, to consider as control before proceeding of molecular docking simulation of all suberitenones (Wang et al., 2010; Greig, 2016). The RMSDs between the experimental poses and the predicted poses were 0.91 Å, 2.36 Å, and 1.87 Å for CASP3, MAPK3, and EGFR, respectively. As an RMSD value ≥3.0 Å is considered unacceptable, the protocol used in this investigation for the reproduction of native structure was good (Ramírez and Caballero, 2018). In the case of 3KJF, suberitenone I showed a similar binding affinity as the control ligand B92 (−8.9 kcal/mol), which is given in Table 5. Secosuberitenone A showed higher binding affinity against 6GES than 6H3 (−9.2 kcal/mol). The most significant finding of this investigation is that suberitenone E and suberitenone J are bound to 6JXT with higher energy than osimertinib, which showed a binding affinity of −8.5 kcal/mol.

As illustrated in Figure 6a, suberitenone I formed one conventional hydrogen bond and one carbon–hydrogen bond (Table 6 **)** with the PHE250 residue in the active site of 3KJF. Although control B92 could form several hydrogen and hydrophobic bonds with different residues (Supplementary Figure S1a), a greater number of bonds does not always give the best net binding affinity. In the case of 6GES, secosuberitenone A could form more hydrogen as well as hydrophobic bonds than the control 6H3 against 6GES, which can account for the higher binding score of secosuberitenone A (Figure 6b; Supplementary Figure S1b). Both suberitenone E and suberitenone J bind to 6JXT by forming different types of hydrophobic bonds (Figures 6c,d). In addition, suberitenone E could form conventional and carbon–hydrogen bonds, followed by one unfavorable acceptor–acceptor interaction (Table 6).

**TABLE 6:** Molecular docking study of different investigational compounds against PDB IDs: 3KJF, 6GES, and 6JXT.

Investigational compounds	Residues in contact	Bond category/type	Distance (Å)
Target (3KJF)			
Suberitenone I	PHE250	Conventional H bond	2.3058
	PHE250	Carbon H bond	3.42109
B92	ARG207	Conventional H bond	1.9761
	SER205	Conventional H bond	2.68364
	SER205	Carbon H Bond	3.35613
	SER209	Conventional H bond	1.93883
	ARG207	Electrostatic/pi-cation	4.38085
	PHE250	Hydrogen Bond/pi-donor H bond	3.23337
	ARG207	Hydrophobic/amide-pi stacked	4.43648
	PHE256	Hydrophobic/pi-alkyl	4.71292
	TYR204	Hydrophobic/pi-alkyl	5.0633
Target (6GES)			
Secocuberitenone A	ALA52	Conventional H bond	2.55989
	TYR53	Conventional H bond	1.73315
	CYS183	Conventional H bond	2.98031
	ALA52	Hydrophobic/alkyl	4.14299
	LYS71	Hydrophobic/alkyl	4.70555
	CYS183	Hydrophobic/alkyl	4.92947
	LEU173	Hydrophobic/alkyl	5.40996
	ILE48	Hydrophobic/alkyl	3.86573
	VAL56	Hydrophobic/alkyl	3.64821
	CYS183	Hydrophobic/alkyl	3.88515
	TYR53	Hydrophobic/pi-alkyl	4.65127
6H3	MET125	Conventional H bond	2.74744
	ASP184	Carbon H bond	3.49354
	GLU50, GLY51	Hydrophobic/amide-pi stacked	4.11341
	TYR53	Hydrophobic/pi-alkyl	4.6239
	LYS71	Hydrophobic/pi-alkyl	5.40093
	VAL56	Hydrophobic/pi-alkyl	4.37612
	ALA69	Hydrophobic/pi-alkyl	5.03039
	LEU173	Hydrophobic/pi-alkyl	4.84719
	CYS183	Hydrophobic/pi-alkyl	5.13799
Target (6JXT)			
Suberitenone E	ALA743	Hydrophobic/alkyl	4.17862
	LEU844	Hydrophobic/alkyl	4.22475
Suberitenone J	SER720	Conventional H bond	2.89436
	CYS797	Conventional H bond	2.76418
Target (6JXT)			
	GLY796	Carbon H bond	3.21557
	LEU718	Hydrophobic/alkyl	5.16139
	ALA743	Hydrophobic/alkyl	4.41549
	LEU844	Hydrophobic/alkyl	4.79319
Osimertinib	CYS797	Conventional H bond	2.52145
	MET793	Conventional H bond	2.78451
	GLN791	Carbon H bond	3.48641
	PRO794	Carbon H bond	3.7597
	GLU804	Carbon H bond	3.69414
	LEU718	Hydrophobic/pi-sigma	3.46085
	VAL726	Hydrophobic/pi-sigma	3.96557
	LEU718	Hydrophobic/alkyl	5.16341
	LEU792	Hydrophobic/alkyl	4.58291
	VAL726	Hydrophobic/pi-alkyl	4.36024
	LYS120	Hydrophobic/pi-alkyl	5.23876

### 3.9 MD simulation analysis

#### 3.9.1 RMSD analysis

**FIGURE 7:**
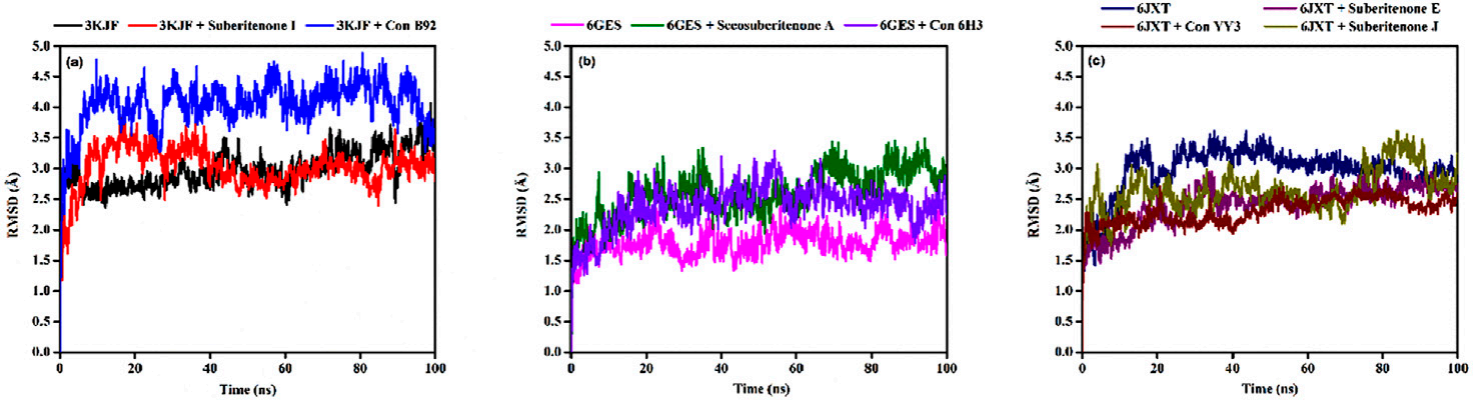
The root means square deviations (RMSDs) of protein–ligand docked complexes. **(a)** The RMSDs of the native protein 3KJF in the absence of a ligand (black) and in the presence of suberitenone I (red) and the RMSD of control ligand B92 (blue). **(b)** The RMSDs of the native protein 6GES in the absence of a ligand (magenta) and in the presence of secosuberitenone A (olive) and the RMSD of the control ligand 6H3 (violet). **(c)** The RMSDs of the native protein 6JXT in the absence of a ligand (navy) and in the presence of suberitenone E (purple) and suberitenone J (dark yellow) and the RMSD of control ligand YY3 (wine).

RMSD is widely used to analyze macromolecular structures by comparing the estimated degree of three-dimensional structural similarity between two or more proteins after optimal superposition. In addition to the total number of atoms included in the structural alignment, dimensions of structures and conformational differences are the key features for measuring RMSD (Carugo and Pongor, 2001; Carugo, 2003). The acceptable RMSD range for the protein–ligand complex is 1–3 Å. An RMSD above 3 Å resonates for the significant conformational changes of the protein during simulation. A 100-ns MD simulation was performed to evaluate the changes in the conformation of 3KJF-suberitenone I, 3 kJ F-Control B92, 6GES-suberitenone A, 6GES-Control 6H3, 6JXT-suberitenone E, 6JXT-suberitenone J, and 6JXT-Control YY3. Initially, the RMSD of suberitenone I showed fluctuation between 2.5 Å and 3.5 Å (Figure 7a). Later, from 40 ns to 100 ns, suberitenone I showed almost no deviation from 3 Å as well as overlapping with the RMSD of 3KJF, which is in apo form. In contrast, control B92 showed a high RMSD of 4.5 Å with several fluctuations from the beginning to 95 ns followed by a decrease in RMSD to 3 Å.

In Figure 7b, the RMSD of the second wild-type apo protein (PDB ID: 6GES) remained stable at 1.8 Å for the entire simulation period. The control 6H3, when bound to 6GES, showed a very stable RMSD of 2.5 Å as well. Although the investigational compound secosuberitenone A completely overlapped with the control 6H3 from the beginning to 70 ns, later, the RMSD went higher to 3 Å and became stable at the end of the simulation period.

In the case of suberitenone E and control YY3 bound to 6JXT, the RMSD gradually increased from 2 Å to 2.5 Å and became stable after 50 ns (Figure 7c). The RMSD of suberitenone J overlapped with suberitenone E and the control YY3 from 50 ns to the end of the simulation period; however, suberitenone J also showed a significant fluctuation from 80 ns to 90 ns.

According to the RMSD analysis, it can be considered that all suberitenones showed very stable and, in some cases, similar or better RMSD values than the control compounds when bound to their respective proteins.

#### 3.9.2 RMSF analysis

**FIGURE 8:**
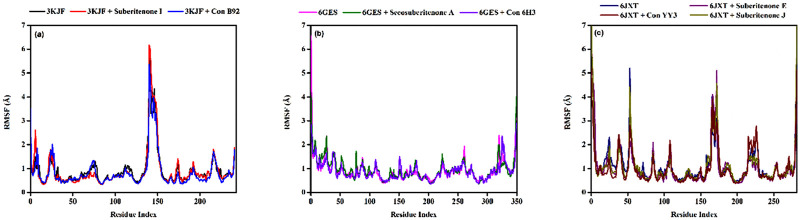
Variation in the root means square fluctuation (RMSF) of protein–ligand docked complexes. **(a)** The RMSFs of the native protein 3KJF in the absence of a ligand (black) and in the presence of suberitenone I (red) and the RMSF of control ligand B92 (blue). **(b)** The RMSF of the native protein 6GES in the absence of a ligand (magenta) and in the presence of secosuberitenone A (olive) and the RMSF of control ligand 6H3 (violet). **(c)** The RMSF of the native protein 6JXT in the absence of a ligand (navy) and in the presence of suberitenone E (purple) and suberitenone J (dark yellow) and the RMSF of control ligand YY3 (wine).

RMSF analysis is used to check the residual fluctuations over the simulation period in protein with or without any ligand and whether the flexible residues are from the active site (Adelusi et al., 2022). The RMSF values of the wild-type protein (PDB ID: 3KJF) were assessed in the presence or absence of suberitenone I and control B92 and demonstrated that the RMSF values of the residues between THR140 to ARG149 were very high not only in the ligand-bound state but also in the case of apo wild-type protein (Figure 8a). In the case of wild-type protein PDB ID: 6GES, no major oscillation was observed due to the binding of either secosuberitenone A or the control 6H3 (Figure 8b).

The RMSF of wild-type protein PDB ID: 6JXT was observed mainly for residues including GLU749, GLY863 to ALA871, and LEU927 (Figure 8c). The fluctuation at residue GLU749 was highly reduced with the addition of suberitenone E to 6JXT. Major fluctuations of RMSF were observed at the region of GLY863 to ALA871 for suberitenone E and suberitenone J when bound to 6JXT. Finally, minor oscillations of residue LEU927 were observed for the control YY3, but the RMSF remained low after the addition of suberitenone E and suberitenone J to 6JXT. As these regions of 6JXT are very far from the catalytic site, no deviation in the binding affinity is expected due to the binding of any of the suberitenones.

#### 3.9.3 Radius of gyration (R_g_)

**FIGURE 9:**
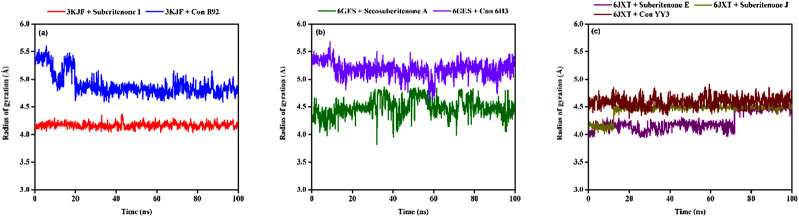
The radius of gyration (R_g_) values of different proteins and ligand complexes were calculated from the 100-ns simulation. **(a)** The R_g_ values of the selected ligands suberitenone I (red) and control B92 (blue) in complex with 3KJF. **(b)** The R_g_ values of the selected ligands secosuberitenone A (olive) and control 6H3 (violet)in complex with 6GES. **(c)** The R_g_ values of the selected ligands suberitenone E (purple), suberitenone (dark yellow), and control YY3 (wine) in complex with 6JXT.

The radius of gyration (R_g_) is a parameter that indicates the compactness of amino acid residues in proteins (Lobanov et al., 2008; Adelusi et al., 2022). The R_g_ values of 3KJF-suberitenone I and 3KJF–Control B92 complexes varied in the range between 4.039 Å to 4.366 Å and 4.581 Å to 5.604 Å with an average of 4.16 ± 0.04 Å and 4.90 ± 0.20 Å, respectively (Figure 9a). According to R_g_ data, suberitenone I showed compact and stable binding with 3 kJ F compared to the control B92. In the case of 6GES-secosuberitenone A and 6GES-Control 6H3 complexes, the R_g_ values fluctuated in a range between 3.814 Å to 4.862 Å and 4.590 Å to 5.674 Å with an average of 4.49 ± 0.16 Å, and 5.18 ± 0.14 Å, respectively (Figure 9b). Although the average R_g_ value of the 6GES-secosuberitenone A complex is lower than that of 6GES-Control 6H3, greater fluctuation makes the investigational compound secosuberitenone A less suitable for binding with 6GES than the control 6H3. The R_g_ values of 6JXT-suberitenone E and 6JXT-suberitenone J overlapped with those of the 6JXT-Control YY3 complex from 75 ns and 15 ns to the end of the simulation, respectively. The 6JXT–Control YY3 complex showed an R_g_ between 4.292 Å and 4.907 Å with an average of 4.59 ± 0.09 Å (Figure 9c). All three complexes showed low fluctuation of R_g_, which confirms the stable binding of those complexes.

#### 3.9.4 Solvent accessible surface area (SASA)

**FIGURE 10:**
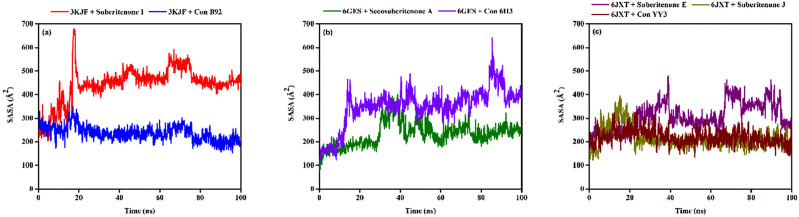
The solvent accessible surface area (SASA) values of different proteins and ligand complexes were calculated from the 100-ns simulation. **(a)** The SASA values of the selected ligands suberitenone I (red) and control B92 (blue) in complex with 3KJF. **(b)** The SASA values of the selected ligands secosuberitenone A (olive) and control 6H3 (violet) in complex with 6GES. **(c)** The SASA values of the selected ligands suberitenone E (purple), suberitenone J (dark yellow), and control YY3 (wine) in complex with 6JXT.

SASA analysis is crucial for checking the surface area susceptible to the solvent, as the increase in the protein–ligand complex’s surface area can lead to the unfolding of the protein (Lobanov et al., 2008). In the case of 3KJF–suberitenone I and 3KJF–Control B92 complexes, the SASA values fluctuated in a range between 201.51 Å^2^ to 680.03 Å^2^ and 153.96 Å^2^ to 349.05 Å^2^ with an average of 443.94 ± 75.45 Å^2^ and 237.05 ± 31.72 Å^2^, respectively (Figure 10a). Although the 3KJF–Control B92 complex showed very low oscillation, the SASA of the 3KJF–Suberitenone I complex was also stable from 20 ns to the end of the simulation period. SASA values for the 6GES–secosuberitenone A and 6GES–Control 6H3 complexes varied in the range of 73.27 Å^2^ to 399.66 Å^2^ and 86.82 Å^2^ to 639.76 Å^2^ with an average of 232.23 ± 51.31 Å^2^ and 350.78 ± 80.23 Å^2^, respectively (Figure 10b). The SASA value of 6GES-secosuberitenone A fluctuated for a small period (30 ns–40 ns) and later became stable for the remaining simulation period. Finally, the SASA plot of 6JXT-suberitenone J completely overlaps with 6JXT-Control YY3, which shows SASA values between 135.23 Å^2^ and 331.05 Å^2^ with an average of 218.92 ± 31.66 Å^2^ (Figure 10c). For 6JXT–suberitenone E, not only the SASA value was higher with an average of 350.78 ± 80.23 Å^2^, but also the overall SASA values ranged between 119.50 Å^2^ and 478.99 Å^2^ with significant fluctuations from 65 ns to 95 ns.

#### 3.9.5 Protein–ligand contact analysis

**FIGURE 11:**
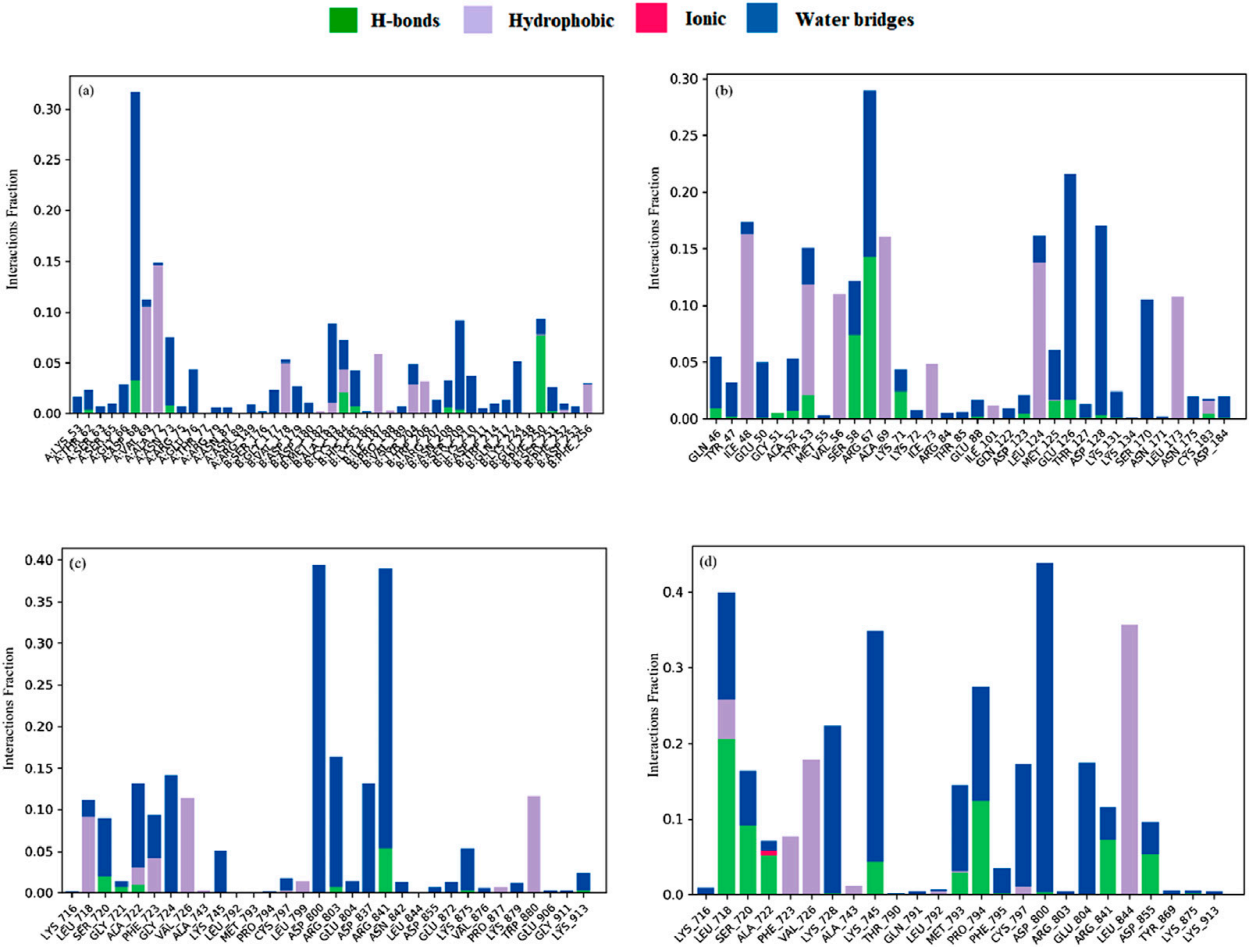
The stacked bar charts represent the protein–ligands interactions found during the 100 ns simulation. Herein, showing the interaction of selected compounds **(a)** suberitenone I in complex with 3KJF; **(b)** secosuberitenone A in complex with 6GES; **(c)** suberitenone E; **(d)** suberitenone J in complex with 6JXT.

Suberitenone I formed considerable hydrophobic interactions with VAL69, ALA72, and ILE187, followed by forming a hydrogen bond with PHE250 with 3KJF (Figure 11a). Suberitenone A bound to 6GES forming hydrophobic interactions with ILE48, TYR53, VAL56, ALA69, LEU124, and LEU173 (Figure 11b). Secosuberitenone A also formed hydrogen bonds with residues SER58 and ARG67 of 6GES. In the case of the 6JXT–suberitenone E complex, LEU718, PHE723, VAL726, and TRP880 were observed to form hydrophobic bonds, and ARG841 was observed to form hydrogen bonds (Figure 11c). Instead of TRP880, suberitenone J formed hydrophobic bonds with the LEU844, and the remaining residues formed the same hydrophobic bonds as suberitenone E (Figure 11d). Suberitenone J formed hydrogen bonds with LEU718, SER720, ALA722, PRO794, ARG841, and ASP855 and formed one ionic bond with ALA722. These results suggest better binding of suberitenone J than suberitenone E with 6JXT.

## 4 Conclusion

Secosuberitenone A, suberitenone E, suberitenone I, and suberitenone J are good drug candidates with CNS penetration ability and BBB, according to the QSAR and ADMET study. CYP3A4 can metabolize these four suberitenones as well. Of them, suberitenone I had the highest affinity for CAP3 (PDB ID: 3KJF), at −8.9 kcal/mol. Two factors that are considered very important when selecting good drug-like candidates are the criteria of Lipinski’s “Rule of Five” and the analysis of toxicity. Suberitenone I not only fulfilled Lipinski’s “Rule of Five” criteria but also exhibited good toxicity properties. Secosuberitenone A attached to MAPK3 (PDB ID: 6GES) with −9.4 kcal/mol binding energy and did not show any types of drawbacks in all the structural analyses performed. Suberitenone E and suberitenone J exhibited a higher binding affinity of −8.8 kcal/mol against one of the five core target EGFRs (PDB ID: 6JXT). These two showed low toxicity properties and met the Lipinski “Rule of Five” criteria. However, the low LD50 values of suberitenone I, suberitenone E, and suberitenone J must be investigated using wet lab analysis. Additionally, both secosuberitenone A and suberitenone J might exhibit low carcinogenicity even if they are found to be antineoplastic, according to PASS prediction. Two distinct web servers were the sources of both traits, which is one of the two explanations for this contradiction. Second, a recent study suggested that antineoplastic drugs (ANDs) may cause secondary cancers in chemotherapy patients, which might also help to explain this effect (Müller-Ramírez et al., 2023). Secosuberitenone A is the safest suberitenone, while suberitenone E was found to be more toxic than the others in the cardiac safety assessment.

The RMSD values indicate that the investigational suberitenones can form stable bonds with their respective proteins. According to the RMSF data, the residues that experienced fluctuations during the simulation were located outside of the catalytic site. Therefore, these fluctuations are unlikely to affect the binding affinities of the protein–ligand complexes. According to R_g_, SASA, and protein–ligand contact analysis, suberitenone I and secosuberitenone A could be considered valid as a novel small molecule that could bind with similar or more stability than the control B92 and control 6H3 with their respective proteins. However, compared to suberitenone J and the control YY3, these analyses also showed that suberitenone E is less suitable for forming a complex with 6JXT. Finally, among all the investigational suberitenones, suberitenone I, secosuberitone A, and suberitenon J have the best drug-like qualities and can become potent inhibitors of CASP3, MAPK3, and EGFR, respectively, based on the results of this *in silico* study. Even though secosuberitenone A, suberitenone E (observed to be a cardiac failure from toxicity studies), suberitenone I, and suberitenone J were good drug candidates according to QSAR, their LD50 values are concerning issues that require additional study and attention before proceeding toward *in vitro* and *in vivo* investigations.

## Data Availability

The original contributions presented in the study are included in the article/Supplementary Material; further inquiries can be directed to the corresponding author.
