# Supplementary material for: Network pharmacology and molecular dynamics simulation reveal antineoplastic potential of Antarctic sponge-derived suberitenones
Source: Front Chem. 2025 May 27;13:1545834. doi: 10.3389/fchem.2025.1545834 (PMC12148877; doi:10.3389/fchem.2025.1545834)
Supplement: Supplementary file 1 [file DataSheet1.docx]

**Supplementary data**

**
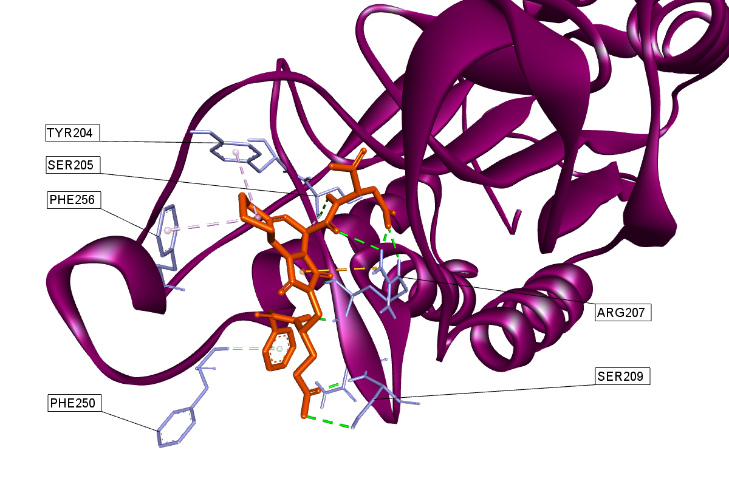

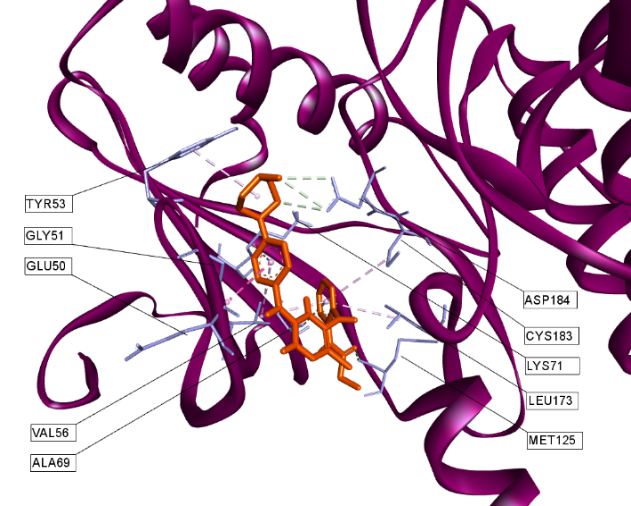
**

1. **(b)**

**
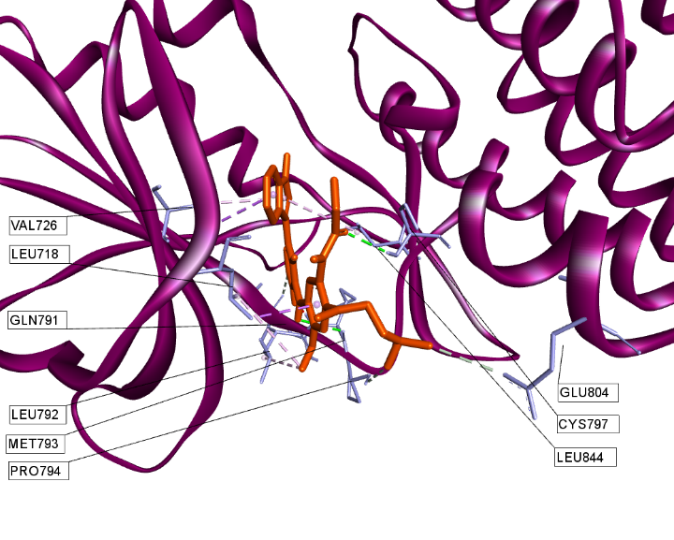
**

**(c)**

**Supplementary Figure 1:** 3D structures of (a) B92, (b) 6H3, and (c) Osimertinib bound to the catalytic site of CASP3 (PDB: 3KJF), MAPK3 (PDB: 6GES), and EGFR (PDB: 6JXT) respectively.

**Supplementary Table 1: Cross-target prediction of Suberitenones and diseases**

| **Target** | **Common name** | **Uniprot ID** |
| --- | --- | --- |
| 11-beta-hydroxysteroid dehydrogenase 1 | HSD11B1 | P28845 |
| Cytochrome P450 19A1 | CYP19A1 | P11511 |
| HMG-CoA reductase (by homology) | HMGCR | P04035 |
| Cyclooxygenase-2 | PTGS2 | P35354 |
| Protein-tyrosine phosphatase 1B | PTPN1 | P18031 |
| Androgen Receptor | AR | P10275 |
| Carboxylesterase 2 | CES2 | O00748 |
| Cytochrome P450 17A1 | CYP17A1 | P05093 |
| Progesterone receptor | PGR | P06401 |
| Prostaglandin E synthase | PTGES | O14684 |
| 11-beta-hydroxysteroid dehydrogenase 2 | HSD11B2 | P80365 |
| LXR-alpha | NR1H3 | Q13133 |
| Trypsin I | PRSS1 | P07477 |
| T-cell protein-tyrosine phosphatase | PTPN2 | P17706 |
| Thrombin | F2 | P00734 |
| Glucocorticoid receptor | NR3C1 | P04150 |
| Prostanoid IP receptor | PTGIR | P43119 |
| Norepinephrine transporter | SLC6A2 | P23975 |
| Neurokinin 2 receptor | TACR2 | P21452 |
| Dopamine transporter | SLC6A3 | Q01959 |
| Mineralocorticoid receptor | NR3C2 | P08235 |
| C5a anaphylatoxin chemotactic receptor | C5AR1 | P21730 |
| Sphingosine 1-phosphate receptor Edg-3 | S1PR3 | Q99500 |
| Beta amyloid A4 protein | APP | P05067 |
| Testis-specific androgen-binding protein | SHBG | P04278 |
| Cannabinoid receptor 1 | CNR1 | P21554 |
| Cannabinoid receptor 2 | CNR2 | P34972 |
| Phospholipase A2 group IIA | PLA2G2A | P14555 |
| Mitogen-activated protein kinase kinase kinase 11 | MAP3K11 | Q16584 |
| Aldo-keto reductase family 1 member C2 | AKR1C2 | P52895 |
| Aldo-keto reductase family 1 member C1 | AKR1C1 | Q04828 |
| Glycogen synthase kinase-3 beta | GSK3B | P49841 |
| Estradiol 17-beta-dehydrogenase 2 | HSD17B2 | P37059 |
| Steroid 5-alpha-reductase 2 | SRD5A2 | P31213 |
| Vascular endothelial growth factor receptor 2 | KDR | P35968 |
| Lysosomal Pro-X carboxypeptidase | PRCP | P42785 |
| MAP kinase p38 alpha | MAPK14 | Q16539 |
| Inhibitor of nuclear factor kappa B kinase beta subunit | IKBKB | O14920 |
| LXR-beta | NR1H2 | P55055 |
| Liver glycogen phosphorylase | PYGL | P06737 |
| Apoptosis regulator Bcl-X | BCL2L1 | Q07817 |
| DNA polymerase beta | POLB | P06746 |
| Receptor-type tyrosine-protein phosphatase F (LAR) | PTPRF | P10586 |
| Phospholipase A2 group 1B | PLA2G1B | P04054 |
| Low molecular weight phosphotyrosine protein phosphatase | ACP1 | P24666 |
| Aldo-keto reductase family 1 member B10 | AKR1B10 | O60218 |
| LDL-associated phospholipase A2 | PLA2G7 | Q13093 |
| Endothelial PAS domain-containing protein 1 | EPAS1 | Q99814 |
| N-lysine methyltransferase SMYD2 | SMYD2 | Q9NRG4 |
| Voltage-gated potassium channel subunit Kv1.5 | KCNA5 | P22460 |
| Anandamide amidohydrolase | FAAH | O00519 |
| Muscarinic acetylcholine receptor M2 | CHRM2 | P08172 |
| Sigma opioid receptor | SIGMAR1 | Q99720 |
| P-glycoprotein 1 | ABCB1 | P08183 |
| Estrogen receptor beta | ESR2 | Q92731 |
| Steryl-sulfatase | STS | P08842 |
| Transient receptor potential cation channel subfamily V member 4 (by homology) | TRPV4 | Q9HBA0 |
| Heat shock protein HSP 90-alpha | HSP90AA1 | P07900 |
| Sphingosine 1-phosphate receptor Edg-1 | S1PR1 | P21453 |
| EZH2/SUZ12/EED/RBBP7/RBBP4 | EZH2 | Q15910 |
| Cathepsin D | CTSD | P07339 |
| Tyrosine-protein kinase JAK2 | JAK2 | O60674 |
| Proto-oncogene tyrosine-protein kinase MER | MERTK | Q12866 |
| Corticosteroid binding globulin | SERPINA6 | P08185 |
| Estradiol 17-beta-dehydrogenase 3 | HSD17B3 | P37058 |
| Fatty acid binding protein adipocyte | FABP4 | P15090 |
| Peroxisome proliferator-activated receptor alpha | PPARA | Q07869 |
| Fatty acid binding protein epidermal | FABP5 | Q01469 |
| Peroxisome proliferator-activated receptor delta | PPARD | Q03181 |
| Free fatty acid receptor 1 | FFAR1 | O14842 |
| Fatty acid binding protein intestinal | FABP2 | P12104 |
| UDP-glucuronosyltransferase 2B7 | UGT2B7 | P16662 |
| Vitamin D receptor | VDR | P11473 |
| Solute carrier family 22 member 6 (by homology) | SLC22A6 | Q4U2R8 |
| Glucose-6-phosphate 1-dehydrogenase | G6PD | P11413 |
| Bile acid receptor FXR | NR1H4 | Q96RI1 |
| Dual specificity phosphatase Cdc25A | CDC25A | P30304 |
| G-protein coupled bile acid receptor 1 | GPBAR1 | Q8TDU6 |
| Lysine-specific demethylase 2A | KDM2A | Q9Y2K7 |
| Histone lysine demethylase PHF8 | PHF8 | Q9UPP1 |
| Lysine-specific demethylase 5C | KDM5C | P41229 |
| Prostanoid EP2 receptor | PTGER2 | P43116 |
| Indoleamine 2,3-dioxygenase | IDO1 | P14902 |
| Plasminogen | PLG | P00747 |
| Hypoxia-inducible factor 1 alpha | HIF1A | Q16665 |
| CDC45-related protein | CDC45 | O75419 |
| Leukocyte common antigen | PTPRC | P08575 |
| Serotonin transporter (by homology) | SLC6A4 | P31645 |
| Carbonic anhydrase II | CA2 | P00918 |
| Carbonic anhydrase I | CA1 | P00915 |
| Transient receptor potential cation channel subfamily M member 8 | TRPM8 | Q7Z2W7 |
| Ileal bile acid transporter | SLC10A2 | Q12908 |
| Voltage-gated calcium channel alpha2/delta subunit 1 | CACNA2D1 | P54289 |
| Vanilloid receptor | TRPV1 | Q8NER1 |
| Epoxide hydratase | EPHX2 | P34913 |
| Beta-secretase 1 | BACE1 | P56817 |
| Nitric oxide synthase, inducible (by homology) | NOS2 | P35228 |
| TNF-alpha | TNF | P01375 |
| Butyrylcholinesterase | BCHE | P06276 |
| Protein kinase C eta | PRKCH | P24723 |
| Arachidonate 5-lipoxygenase | ALOX5 | P09917 |
| Squalene synthetase (by homology) | FDFT1 | P37268 |
| Protein-tyrosine phosphatase 2C | PTPN11 | Q06124 |
| Adenosine A3 receptor | ADORA3 | P0DMS8 |
| MAP kinase ERK1 | MAPK3 | P27361 |
| Estrogen receptor alpha | ESR1 | P03372 |
| Nuclear receptor ROR-alpha | RORA | P35398 |
| N-lysine methyltransferase SETD8 | KMT5A | Q9NQR1 |
| C-C chemokine receptor type 1 | CCR1 | P32246 |
| Prostanoid EP1 receptor | PTGER1 | P34995 |
| Pregnane X receptor | NR1I2 | O75469 |
| PI3-kinase p110-beta subunit | PIK3CB | P42338 |
| Interleukin-6 receptor subunit beta | IL6ST | P40189 |
| Period circadian protein homolog 2 | PER2 | O15055 |
| Mu opioid receptor (by homology) | OPRM1 | P35372 |
| Delta opioid receptor (by homology) | OPRD1 | P41143 |
| Kappa Opioid receptor (by homology) | OPRK1 | P41145 |
| Peroxisome proliferator-activated receptor gamma | PPARG | P37231 |
| P2X purinoceptor 3 | P2RX3 | P56373 |
| Cell division protein kinase 8 | CDK8 | P49336 |
| Serine/threonine-protein kinase RIPK2 | RIPK2 | O43353 |
| Tyrosine-protein kinase FYN | FYN | P06241 |
| Tyrosine-protein kinase YES | YES1 | P07947 |
| Serine/threonine-protein kinase Aurora-B | AURKB | Q96GD4 |
| Tyrosine-protein kinase SRC | SRC | P12931 |
| Serine/threonine-protein kinase Aurora-A | AURKA | O14965 |
| Prostanoid EP4 receptor | PTGER4 | P35408 |
| dUTP pyrophosphatase | DUT | P33316 |
| Cytochrome P450 51 | CYP51A1 | Q16850 |
| Serotonin 1a (5-HT1a) receptor | HTR1A | P08908 |
| Matrix metalloproteinase 3 | MMP3 | P08254 |
| Matrix metalloproteinase 9 | MMP9 | P14780 |
| Matrix metalloproteinase 1 | MMP1 | P03956 |
| Terminal deoxynucleotidyltransferase | DNTT | P04053 |
| Phosphodiesterase 2A | PDE2A | O00408 |
| Renin | REN | P00797 |
| Phosphodiesterase 10A | PDE10A | Q9Y233 |
| Poly [ADP-ribose] polymerase-1 | PARP1 | P09874 |
| Hormone sensitive lipase | LIPE | Q05469 |
| Sodium channel protein type IX alpha subunit | SCN9A | Q15858 |
| MAP kinase ERK2 | MAPK1 | P28482 |
| Centromere-associated protein E | CENPE | Q02224 |
| Tyrosine-protein kinase JAK3 | JAK3 | P52333 |
| Tyrosine-protein kinase JAK1 | JAK1 | P23458 |
| Tyrosine-protein kinase TYK2 | TYK2 | P29597 |
| Proteinase-activated receptor 1 | F2R | P25116 |
| Prostanoid EP3 receptor | PTGER3 | P43115 |
| Adenosine deaminase | ADA | P00813 |
| Polyadenylate-binding protein 1 | PABPC1 | P11940 |
| Cyclin-dependent kinase 2 | CDK2 | P24941 |
| Protein kinase C delta | PRKCD | Q05655 |
| Protein kinase C gamma | PRKCG | P05129 |
| Protein kinase C alpha | PRKCA | P17252 |
| Protein kinase C beta | PRKCB | P05771 |
| Protein kinase C epsilon | PRKCE | Q02156 |
| Sphingosine 1-phosphate receptor Edg-5 | S1PR2 | O95136 |
| Tubulin--tyrosine ligase | TTL | Q8NG68 |
| Aldo-keto-reductase family 1 member C3 | AKR1C3 | P42330 |
| DNA polymerase alpha subunit | POLA1 | P09884 |
| Dual specificity protein kinase TTK | TTK | P33981 |
| Dual specificity phosphatase Cdc25C | CDC25C | P30307 |
| RAS guanyl-releasing protein 1 (by homology) | RASGRP1 | O95267 |
| Proto-oncogene vav | VAV1 | P15498 |
| Neuropeptide Y receptor type 5 (by homology) | NPY5R | Q15761 |
| Cholecystokinin B receptor | CCKBR | P32239 |
| Macrophage colony stimulating factor receptor | CSF1R | P07333 |
| Stem cell growth factor receptor | KIT | P10721 |
| Type-1 angiotensin II receptor (by homology) | AGTR1 | P30556 |
| C-C chemokine receptor type 8 | CCR8 | P51685 |
| Mitogen-activated protein kinase kinase kinase 5 | MAP3K5 | Q99683 |
| ALK tyrosine kinase receptor | ALK | Q9UM73 |
| Serine/threonine-protein kinase mTOR | MTOR | P42345 |
| PI3-kinase p110-alpha subunit | PIK3CA | P42336 |
| Lymphocyte differentiation antigen CD38 | CD38 | P28907 |
| Protein kinase C theta | PRKCQ | Q04759 |
| Leukocyte adhesion glycoprotein LFA-1 alpha | ITGAL | P20701 |
| Leukocyte elastase | ELANE | P08246 |
| Endoplasmin | HSP90B1 | P14625 |
| Hexokinase type IV | GCK | P35557 |
| Cathepsin L | CTSL | P07711 |
| Heat shock protein HSP 90-beta | HSP90AB1 | P08238 |
| Cyclin-dependent kinase 1 | CDK1 | P06493 |
| C-X-C chemokine receptor type 3 | CXCR3 | P49682 |
| Voltage-gated N-type calcium channel alpha-1B subunit | CACNA1B | Q00975 |
| Tyrosine-protein kinase receptor UFO | AXL | P30530 |
| Tyrosine-protein kinase receptor TYRO3 | TYRO3 | Q06418 |
| C-C chemokine receptor type 9 | CCR9 | P51686 |
| Corticotropin releasing factor receptor 1 | CRHR1 | P34998 |
| Calpain 2 | CAPN2 | P17655 |
| Neurokinin 1 receptor | TACR1 | P25103 |
| Calpain 1 | CAPN1 | P07384 |
| Corticotropin releasing factor receptor 2 | CRHR2 | Q13324 |
| Vesicular acetylcholine transporter | SLC18A3 | Q16572 |
| Prolyl endopeptidase | PREP | P48147 |
| Cytochrome P450 2C19 | CYP2C19 | P33261 |
| Adenosine A2a receptor | ADORA2A | P29274 |
| Proteinase-activated receptor 2 | F2RL1 | P55085 |
| Telomerase reverse transcriptase | TERT | O14746 |
| 6-phosphofructo-2-kinase/fructose-2,6-bisphosphatase 3 | PFKFB3 | Q16875 |
| LIM domain kinase 2 | LIMK2 | P53671 |
| Complement factor D | CFD | P00746 |
| Tyrosine-protein kinase SYK | SYK | P43405 |
| Inhibitor of NF-kappa-B kinase (IKK) | CHUK | O15111 |
| Cytochrome P450 11B1 | CYP11B1 | P15538 |
| Isocitrate dehydrogenase [NADP] cytoplasmic | IDH1 | O75874 |
| Cytochrome P450 11B2 | CYP11B2 | P19099 |
| Tyrosine-protein kinase LCK | LCK | P06239 |
| Nerve growth factor receptor Trk-A | NTRK1 | P04629 |
| Mitogen-activated protein kinase kinase kinase 9 | MAP3K9 | P80192 |
| Mitogen-activated protein kinase kinase kinase 10 | MAP3K10 | Q02779 |
| Proto-oncogene c-JUN | JUN | P05412 |
| Serotonin 2a (5-HT2a) receptor | HTR2A | P28223 |
| Cyclin-dependent kinase 9 | CDK9 | P50750 |
| p53-binding protein Mdm-2 | MDM2 | Q00987 |
| Phosphodiesterase 4D | PDE4D | Q08499 |
| c-Jun N-terminal kinase 1 | MAPK8 | P45983 |
| MAP kinase p38 beta | MAPK11 | Q15759 |
| c-Jun N-terminal kinase 2 | MAPK9 | P45984 |
| Dual specificity phosphatase Cdc25B | CDC25B | P30305 |
| Oxytocin receptor | OXTR | P30559 |
| Mixed lineage kinase 7 | MAP3K20 | Q9NYL2 |
| TGF-beta receptor type II | TGFBR2 | P37173 |
| TGF-beta receptor type I | TGFBR1 | P36897 |
| Sphingosine kinase 2 | SPHK2 | Q9NRA0 |
| Sphingosine kinase 1 | SPHK1 | Q9NYA1 |
| Thrombin and coagulation factor X | F10 | P00742 |
| Serine/threonine protein phosphatase 2A, catalytic subunit, alpha isoform | PPP2CA | P67775 |
| Ceramide glucosyltransferase | UGCG | Q16739 |
| Pyruvate kinase isozymes M1/M2 | PKM | P14618 |
| Dual-specificity tyrosine-phosphorylation regulated kinase 1A | DYRK1A | Q13627 |
| c-Jun N-terminal kinase 3 | MAPK10 | P53779 |
| Scavenger receptor class B member 1 (by homology) | SCARB1 | Q8WTV0 |
| Tyrosine-protein kinase ITK/TSK | ITK | Q08881 |
| Hepatocyte growth factor receptor | MET | P08581 |
| Galanin receptor 1 (by homology) | GALR1 | P47211 |
| Galanin receptor 2 (by homology) | GALR2 | O43603 |
| Rho-associated protein kinase 2 | ROCK2 | O75116 |
| Fatty acid synthase | FASN | P49327 |
| Acetylcholinesterase | ACHE | P22303 |
| Fibroblast activation protein alpha (by homology) | FAP | Q12884 |
| Leucine-rich repeat serine/threonine-protein kinase 2 | LRRK2 | Q5S007 |
| NAD-dependent deacetylase sirtuin 2 | SIRT2 | Q8IXJ6 |
| Serine/threonine-protein phosphatase | PPP5C | Q9BPW0 |
| Smoothened homolog | SMO | Q99835 |
| Sodium/glucose cotransporter 1 | SLC5A1 | P13866 |
| Serine/threonine protein phosphatase PP1-gamma catalytic subunit | PPP1CC | P36873 |
| Bradykinin B1 receptor | BDKRB1 | P46663 |
| Sodium channel protein type V alpha subunit | SCN5A | Q14524 |
| Serine/threonine protein phosphatase PP1-alpha catalytic subunit | PPP1CA | P62136 |
| Tyrosine-protein kinase ABL | ABL1 | P00519 |
| Mitogen-activated protein kinase kinase kinase 14 | MAP3K14 | Q99558 |
| Sorbitol dehydrogenase | SORD | Q00796 |
| Acyl-CoA desaturase | SCD | O00767 |
| Epidermal growth factor receptor erbB1 | EGFR | P00533 |
| Caspase-3 | CASP3 | P42574 |
| Caspase-7 | CASP7 | P55210 |
| Inosine-5'-monophosphate dehydrogenase 2 | IMPDH2 | P12268 |
| Fibroblast growth factor receptor 1 | FGFR1 | P11362 |
| Glycine transporter 2 | SLC6A5 | Q9Y345 |
| Phosphodiesterase 5A | PDE5A | O76074 |
| Rho-associated protein kinase 1 | ROCK1 | Q13464 |
| Ribosomal protein S6 kinase 1 | RPS6KB1 | P23443 |
| Insulin receptor | INSR | P06213 |
| Peptide N-myristoyltransferase 1 | NMT1 | P30419 |
| MAP kinase-activated protein kinase 2 | MAPKAPK2 | P49137 |
| Casein kinase I delta | CSNK1D | P48730 |
| DNA topoisomerase I | TOP1 | P11387 |
| Caspase-6 | CASP6 | P55212 |
| Caspase-8 | CASP8 | Q14790 |
| Caspase-1 | CASP1 | P29466 |
| Interleukin-1 beta | IL1B | P01584 |
| Glutathione S-transferase Mu 1 | GSTM1 | P09488 |
| Voltage-gated potassium channel subunit Kv1.3 | KCNA3 | P22001 |
| Adenosine A2b receptor | ADORA2B | P29275 |
| Alpha-1d adrenergic receptor | ADRA1D | P25100 |
| Alpha-1a adrenergic receptor | ADRA1A | P35348 |
| CDC7/DBF4 (Cell division cycle 7-related protein kinase/Activator of S phase kinase) | CDC7 | O00311 |
| Sarcoplasmic/endoplasmic reticulum calcium ATPase 1 | ATP2A1 | O14983 |
| Dopamine D1 receptor | DRD1 | P21728 |
| HERG | KCNH2 | Q12809 |
| Beta-galactoside alpha-2,6-sialyltransferase 1 | ST6GAL1 | P15907 |
| Cyclooxygenase-1 | PTGS1 | P23219 |
| Von Hippel-Lindau disease tumor suppressor/Elongin B/Elongin C | VHL | P40337 |
| Protein kinase C (PKC) | PRKCZ | Q05513 |
| Muscle glycogen synthase | GYS1 | P13807 |
